# Targeted cellular micropharmacies deliver therapeutic agents to the brain

**DOI:** 10.1038/s44321-026-00421-9

**Published:** 2026-04-14

**Authors:** Manish Malviya, Subha Baniya, Eitan Wong, Tanya Jain, Branavan Manoranjan, Kristen C Vogt, Zoe Kehs, Pedro C Silberman, Tao Dao, Yueming Li, David A Scheinberg

**Affiliations:** 1 Molecular Pharmacology Program, Memorial Sloan-Kettering Cancer Center, New York, NY USA; 2 Gerstner Sloan Kettering Graduate School, Memorial Sloan-Kettering Cancer Center, New York, NY USA; 3 Chemical Biology Program, Memorial Sloan-Kettering Cancer Center, New York, NY USA; 4 Program in Neurosciences, Weill Graduate School of Medical Sciences of Cornell University, New York, NY USA; 5 Human Oncology & Pathogenesis Program, Memorial Sloan-Kettering Cancer Center, New York, NY USA; 6 Tri-institutional Program in Chemical Biology Weill Graduate School of Medical Sciences of Cornell University, New York, NY USA; 7 Program in Pharmacology, Weill Graduate School of Medical Sciences of Cornell University, New York, NY USA; 8 Present Address: R&D Protein Engineering & Novel Modalities; Biologics Engineering, AstraZeneca, 1 MedImmune Way, Gaithersburg, MD USA

**Keywords:** Biotechnology & Synthetic Biology, Methods & Resources, Neuroscience

## Abstract

The systemic administration of therapeutic agents, particularly large, charged molecules such as antibodies, has limited efficacy in treating central nervous system (CNS) disorders. In addition, the slow progression of neurodegenerative diseases makes repeated intrathecal injections unfeasible. Alzheimer’s disease is characterized by the accumulation of Aβ amyloid plaques. Microglia contribute to the clearance of Aβ, but are inhibited by the expression of CD33. Therefore, antibody blocking of CD33 may enhance the phagocytosis of Aβ by microglial cells, slowing AD progression. Here, we use cells as “targeted cellular micropharmacies” that are retained in the CNS to deliver therapeutic proteins directly into the brain. To achieve this, we genetically engineered CD4 T-cells to express: (1) a chimeric antigen receptor against GD2 to retain the cells in the brain, (2) ectopic FoxP3 to reduce inflammation, (3) secreted IL-2 to promote cell longevity, and (4) secreted anti-CD33 scFv antibody. Our proof-of-concept demonstrates that therapeutic antibodies can be delivered to the brain for at least 8 weeks to treat neurological disorders. Other agents could be similarly delivered into the brain by this platform.

## Synopsis

Amyloid-beta (Aβ) aggregates in the brain contribute to Alzheimer’s disease pathogenesis by suppressing phagocytotic function. We have engineered a disialoganglioside (GD2)-specific Treg like CAR-FoxP3 CD4 + T cells capable of infiltrating the CNS and secreting therapeutic anti-CD33 scFv blocking inhibitory CD33 signaling on microglia.

- GD2-specific CD4 + CAR T cells can be reprogrammed into T-reg like cells via ectopic FOXP3 expression, enabling their use as CNS-targeted drug delivery vehicles.
- These engineered Treg-like CAR-FoxP3 CD4 + T cells proliferate in response to neuronal GD2 antigen and autocrine IL-2, thereby allowing sustained secretion of therapeutic anti-CD33 scFv in the CNS for up to eight weeks.
- Anti-CD33 scFv blocks the CD33 receptor on microglia and brain-borne macrophages, enhancing Aβ phagocytosis in peritoneal models.

## Abstract (web summary)

Amyloid-beta (Aβ) aggregates in the brain contribute to Alzheimer’s disease pathogenesis by suppressing phagocytotic function. We have engineered a disialoganglioside (GD2)-specific Treg like CAR-FoxP3 CD4 + T cells capable of infiltrating the CNS and secreting therapeutic anti-CD33 scFv blocking inhibitory CD33 signaling on microglia.

## Graphical abstract

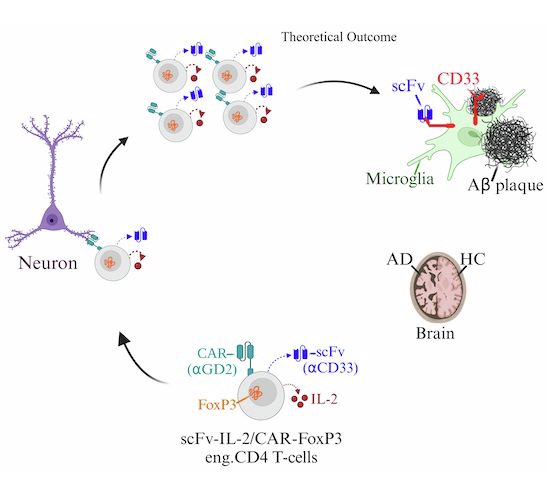

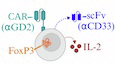

The paper explained **Problem**

Treating brain disorders is particularly challenging because large, charged therapeutics like antibodies cannot efficiently cross the blood-brain barrier (BBB). Existing treatment approaches are often invasive, requiring multiple direct injections into the cerebrospinal fluid. Alzheimer’s disease (AD) is a progressive neurodegenerative disorder that is marked by the buildup of amyloid-beta (Aβ) plaques in the brain. Normally, specialized immune cells in the brain called microglia help remove the plaques. However, these plaques can suppress microglial phagocytic function by engaging with their inhibitory receptor, CD33. Together, this highlights the need for a therapeutic strategy that can both efficiently cross the BBB and restore microglial phagocytic function.

**Results**

In our study, we engineered CD4 T-cells capable of infiltrating the CNS to locally deliver an anti-CD33 single-chain fragment variable (scFv), thereby blocking CD33 interaction with Aβ plaques and potentially mitigating AD-associated pathology. To promote CNS retention, we modified the cells to express a chimeric antigen receptor (CAR) targeting GD2, an antigen expressed in the CNS. We further induced a regulatory phenotype in these T cells to reduce inflammation via ectopic FoxP3 expression and co-expressed IL-2 to enhance cell persistence. Finally, the engineered cells were designed to secrete the therapeutic anti-CD33 scFv. We demonstrate that these Treg-like CAR FoxP3 CD4^+^ T cells infiltrate the brain, adopt an immunosuppressive phenotype, and persist and secrete anti-CD33 scfv for up to 8 weeks in mice. Moreover, in peritoneal models, these engineered T-reg-like CAR T cells enhanced Aβ phagocytosis.

**Impact**

This study establishes a potential cell-based therapeutic platform for neurological diseases by overcoming two major barriers, CNS infiltration and sustained therapeutic delivery. The methods also provide a framework for using engineered immune cells as programmable CNS-targeted drug delivery vehicles that can be applicable to other neurological diseases as well.

## Introduction

The delivery of therapeutic agents into the brain, particularly larger charged molecules and antibodies, is generally inadequate and has limited efficacy (Zhao, 2019; Kouhi et al, 2021; Tian Hui Kwan et al, 2020; Mahase, 2021; Julku et al, 2022). In addition, the slow progression of many neurodegenerative diseases over several years to decades makes repeated intrathecal injections unfeasible. Previously, we have engineered “targeted cellular micropharmacies” to deliver therapeutic proteins, isotopes, or drugs in situ at specific locations in the recipient (Gardner et al, 2020, 2022a; Dacek et al, 2023; Kurtz et al, 2023; Mun et al, 2023; Peraro et al, 2021).

Recently, Lecanemab (Leqembi®) and Donanemab (Kisunla), which target Aβ aggregates and plaques, have been approved by the FDA for early Alzheimer’s Disease (AD) (Sims et al, 2023; van Dyck et al, 2023), indicating that reducing Aβ is a promising treatment for AD. However, the clinical effect of both antibodies in early AD is limited (Sims et al, 2023; van Dyck et al, 2023). In addition, antibodies administered peripherally penetrate the blood-brain barrier (BBB) at a rate of less than 0.1% (Kariolis et al, 2020). Developing novel strategies to deliver biologics to the brain has become a priority in neurodegenerative disorder research.

Genetic studies have identified two genes, TREM2 and CD33, expressed in microglia, that are associated with late-onset Alzheimer’s Disease (LOAD) (Bertram et al, 2008; Jonsson et al, 2013; Guerreiro et al, 2013). Furthermore, TREM2 and CD33 play an important role in the clearance and uptake of Aβ (Griciuc et al, 2013; Wang et al, 2015; Ulrich and Holtzman, 2016). We have shown that Lintuzumab (HuM195) and its scFv variant, which specifically targets CD33, enhance the phagocytosis of Aβ42 in monocytes, macrophages, and microglia (Wong et al, 2024), offering a unique system for developing targeted cellular micropharmacies for AD.

We hypothesized that cells engineered to be retained in the CNS and stably secrete anti-CD33 could be used to deliver the antibody directly to the brain. To this end, we propose using a chimeric antigen receptor (CAR) engineered T-cell that can infiltrate and persist in the brain and secrete an scFv form of HuM195 antibody locally. While the disialoganglioside GD2, an antigen found throughout the brain, could serve as a suitable target, recent work found that CAR T-cells against GD2 led to fatal neurotoxicity due to on-target reactivity (Lammie et al, 1993; Cook et al, 2023; Epperla et al, 2023; Velasco et al, 2023). Hence, GD2 CAR-engineered regulatory CD4 T-cells (CAR Tregs), which are not inflammatory, would be the better choice for this purpose. However, the low percentage of circulating Tregs in the blood and the lack of cell surface-specific markers make it difficult to get enough natural Tregs for engineering purposes. Hence, to reduce the potential toxicity of our scFv-secreting GD2 CAR T-cells, we decided to use minimally cytotoxic CD4 T-cells and further engineered them into less toxic T-regulatory-like CD4 T-cells by introducing an exogenous FoxP3 gene. Exogenous FoxP3 expression has been shown to drive CD4 T-cells into regulatory-like T-cells (Herzog et al, 2019). To promote their longevity, we endowed them with the ability to secrete autocrine IL-2 (Kelly et al, 2002).

To achieve this goal, we genetically engineered CD4 T-cells into Treg-like CAR-FoxP3 CD4 T-cells that express a cell surface CAR against GD2, an exogenous FoxP3, and also secrete IL-2 and anti-CD33 scFv. These Treg-like CAR-FoxP3 engineered CD4 T-cells adopt an immunosuppressive phenotype, infiltrate and show prolonged persistence in the brain, and secrete the scFv locally to block CD33 on microglia and brain-borne macrophages, promoting an anti-inflammatory environment that enables tissue repair. The engineered Treg-like CAR T-cells enhanced Aβ phagocytosis in peritoneal models. This approach enables the prolonged delivery of therapeutic cargo inside the BBB using engineered T cells to treat AD, and potentially a variety of other neurodegenerative diseases and cancers. We propose utilizing engineered cells to accumulate in the central nervous system to deliver biologic therapies directly into the brain. For this purpose, such cells must be engineered to be retained in the brain, to be nontoxic and active over long periods, and to secrete the therapeutic agent stably. Here, we demonstrate such a proof-of-concept with a therapeutic antibody as a potential strategy for AD.

## Results

### Engineered human CD4 T-cells secrete active HuM195 scFv that binds to CD33

**Figure 1:**
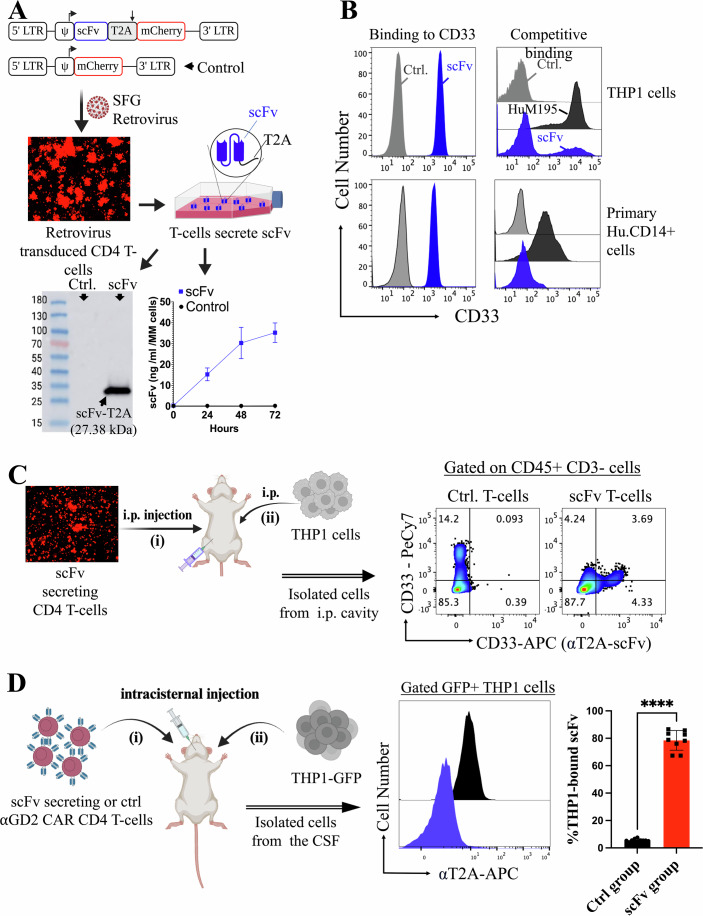
CD4 T-cell transduction and characterization of secreted anti-CD33 scFv antibody. (**A**) Retroviral vector genome: the anti-CD33 scFv gene (VH-(G4S)_3_-VL) was inserted in frame into an SFG-*γ* retroviral vector together with the mCherry gene as a fluorescent marker. The T2A peptide sequence was used to link the scFv and mCherry genes. The control retroviral vector carries only the mCherry gene. A representative image showing CD4 T-cells transduced with retrovirus become mCherry positive (red cells) and secrete scFv antibody in the supernatant that was detected by Western blot and ELISA using an anti-2A peptide antibody. LTR = long-term repeat, Ψ = psi packaging element, CD33 scFv = CD33-specific single chain variable fragment, T2A = 2A self-cleaving peptide, mCherry = red fluorescent protein. (**B**) Representative histograms show the binding of cell-secreted scFv to CD33 on THP1 cells and primary human monocytes (blue left panels). Control cells (gray) did not secrete scFv. The scFv binding to CD33 was abolished in a competition assay where the CD33 receptor was pre-blocked with HuM195 antibody (right panels: black histogram is binding of HuM195 to cells; blue histograms show binding of scFv to CD33 to the same cells after pre-blocking with HuM195), confirming scFv specificity to the CD33 receptor. (**C**) In vivo secretion of scFv and its binding to CD33 was confirmed in live animals in the peritoneal cavity of mice. Two million control or scFv-secreting CD4 T-cells were injected intraperitoneally (i.p.) in mice. Forty-eight hours after T-cell injection, one million THP1 cells were injected into the peritoneal cavity. Thirty minutes later, cells were isolated and analyzed for the THP1 cell-bound scFv by flow cytometry using an anti-2A peptide antibody (αT2A-scFv). A clear population of scFv-bound THP1 cells was observed with an APC-labeled anti-2A peptide antibody. Simultaneously, total CD33 staining was decreased with a commercial PeCy7-labeled anti-CD33 antibody, confirming scFv-masked CD33 in vivo. Representative image of three independent experiments. (**D**) Secretion of scFv by engineered CD4 T cells and binding of the antibody to CD33 were also confirmed in the mouse CNS. 3 million control or scFv secreting αGD2 CAR T cells were injected intracisternaly in NSG mice. Forty-eight hours later, 2 million THP1 cells expressing GFP were also injected into the mice intracisternally. An hour later, brains were harvested and rinsed with PBS to isolate the cells from the CSF. THP1-cell-bound scFV was then analyzed via flow cytometry for both groups using the APC-labeled anti-2A peptide antibody. One example of nine replicates shown with identical results. The bar graph on the right shows the mean ± SD of %THP1-bound scFv across all nine mice for both groups. Statistical significance was calculated using paired *t* test analysis on GraphPad Prism 10 (****P < 0.0001).

An anti-CD33 scFv (VH-(G4S)_3_-VL) retroviral construct was generated from the nucleotide sequences of paired variable regions of HuM195 (Lintuzumab) and cloned into an SFG-*γ* retroviral vector (Rivière et al, 1995). An mCherry gene was also inserted into the scFv construct following a self-cleaving T2A peptide linker sequence to act as a marker of viral transduction (Fig. 1A, upper panel). A retroviral vector with only the mCherry gene was also synthesized as a control. The retroviruses produced by H29/Galv9 producer systems (Gardner et al, 2022b; Jaspers et al, 2023) were used to transduce primary human CD4 T-cells isolated from peripheral blood mononuclear cells (PBMC). The intracellular expression of mCherry confirmed the retrovirus transduction and was used to quantify transduction efficiency by flow cytometry and fluorescence microscopy (Fig. 1A, middle panel). In total, 70–90% transduction of CD4 T-cells was obtained with scFv-mCherry retrovirus (Appendix Fig. S1). We confirmed the cell secretion of the scFv using an anti-2A peptide antibody, which can recognize the first 17 amino acids of the T2A peptide attached to the C-terminal end of the scFv after cleavage (Shibuta et al, 2019). An expected scFv-specific 27 kDa protein band was detected in the western blot from the supernatant of the transduced CD4 T-cells (Fig. 1A, lower left panel). The cumulative release of scFv from the mCherry+ FACS-purified CD4 T-cells was quantified by ELISA to be 40 ng/mL per one million cells after 72 h of culture in serum-free media (Fig. 1A, lower right panel).

Next, we assessed the binding ability of the cell-secreted scFv to its cognate receptor, CD33, on the human leukemia monocytic cell line THP1, and on primary human CD14+ monocytes isolated from PBMCs. Flow cytometry analysis confirmed binding of the CD4 T-cell-secreted scFv on both THP1 cells and monocytes (Fig. 1B, left panels). A competitive binding assay with HuM195 resulted in decreased scFv binding to CD33, confirming that the T-cell- secreted scFv shares the same binding epitope as the parent HuM195 antibody (Fig. 1B, right panels).

To determine if the scFv secreted by engineered CD4 T-cells can bind to monocytes in vivo, we injected mice with two million control or scFv-secreting CD4 T-cells intraperitoneally (i.p.). THP1 monocyte cells were then injected i.p. 48 h later to allow for CD4 T-cell secreted scFv to bind to CD33 on THP1 cells (Fig. 1C). Thirty minutes later, cells were isolated from the peritoneal cavity and analyzed for CD33-bound scFv on THP1 cells. Ex vivo flow cytometry staining confirmed in vivo scFv binding to CD33, which was not detected on THP1 cells isolated from the control T-cell treated group (Fig. 1C). Direct CD33 staining of the THP1 cells using a commercial anti-CD33 antibody was abolished on scFv-secreting T-cell treated group but not on the control group, confirming that in vivo CD33-bound scFv blocked cell surface CD33 (Fig. 1C).

We further tested whether the αCD33 scFv secreted by engineered CD4 T cells can also bind to its target inside the mouse brain. We injected NSG mice (*n* = 9 per group) with 3 million control or scFv-secreting αGD2 CAR CD4 T-cells intracisternally. Forty-eight hours later, respective groups of mice were injected intracisternally with 3 million GFP + THP1 monocytic cells, as a surrogate for human glia.

THP1 cells were isolated from the CSF, and flow cytometry with anti-T2 staining confirmed that the secreted scFv bound to CD33 expressed on THP1 cells inside the brain (Fig. 1D). These results demonstrate that engineered CD4 T-cells can effectively secrete scFv that can bind to CD33 inside the CNS.

### Blocking of CD33 on macrophages and microglia by anti-CD33 scFv induces Aβ42 uptake

**Figure EV1:**
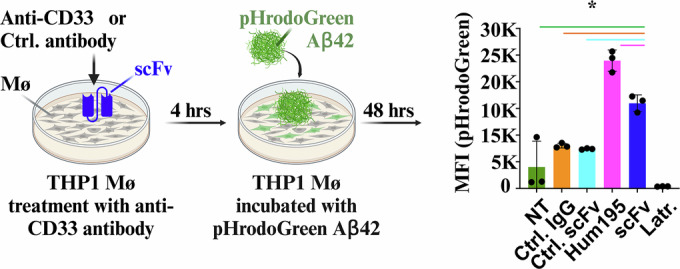
Anti-CD33 scFv treatment increased Aβ42 uptake by THP1 cell-derived macrophages. THP1 cell-derived macrophages (THP1-Mø) were generated as described in materials and methods and were pre-treated with: PBS (no treatment; NT), purified anti-CD33 scFv (1 µg/ml), positive control HuM195 (1 µg/ml), control scFv (1 µg/ml), control IgG (1 µg/ml), and Latrunculin as a negative control (1 µM), respectively, for 4 h before pHrodoGreen-labeled Aβ42 (1 µM) was added to the cells to initiate phagocytosis. Forty-eight hours later, cells were detached, and quantification of phagocytosed pHrodoGreen-Aβ42 was analyzed by flow cytometry. The mean fluorescence intensity (MFI) of pHrodoGreen-positive THP1-Mø is presented in the bar diagram. The two-way ANOVA multiple comparisons statistical analysis was performed using Prism 10. means ± SEM; *n* = 3 (*P < 0.05).

Next, we investigated whether blocking CD33 by CD4 T-cell secreted scFv supernatant fluid or purified scFv on THP1 cell-derived macrophages (THP1-Mø) and primary human macrophages (Hu-Mø) can enhance their Aβ42 phagocytic activity. We utilized a “pHrodo” dye-labeled Aβ42 to track their phagocytosis. pHrodo is a pH-sensitive dye that emits a fluorescent signal when internalized to low-pH lysosomes that can be analyzed by fluorescence microscopy and flow cytometry (Lindner et al, 2020). We observed a significant increase in pHrodo-Aβ42 uptake by both anti-CD33 scFv and HuM195 pre-treated THP1-Mø compared to those pre-treated with control scFv or IgG (Fig. EV1).

**Figure 2:**
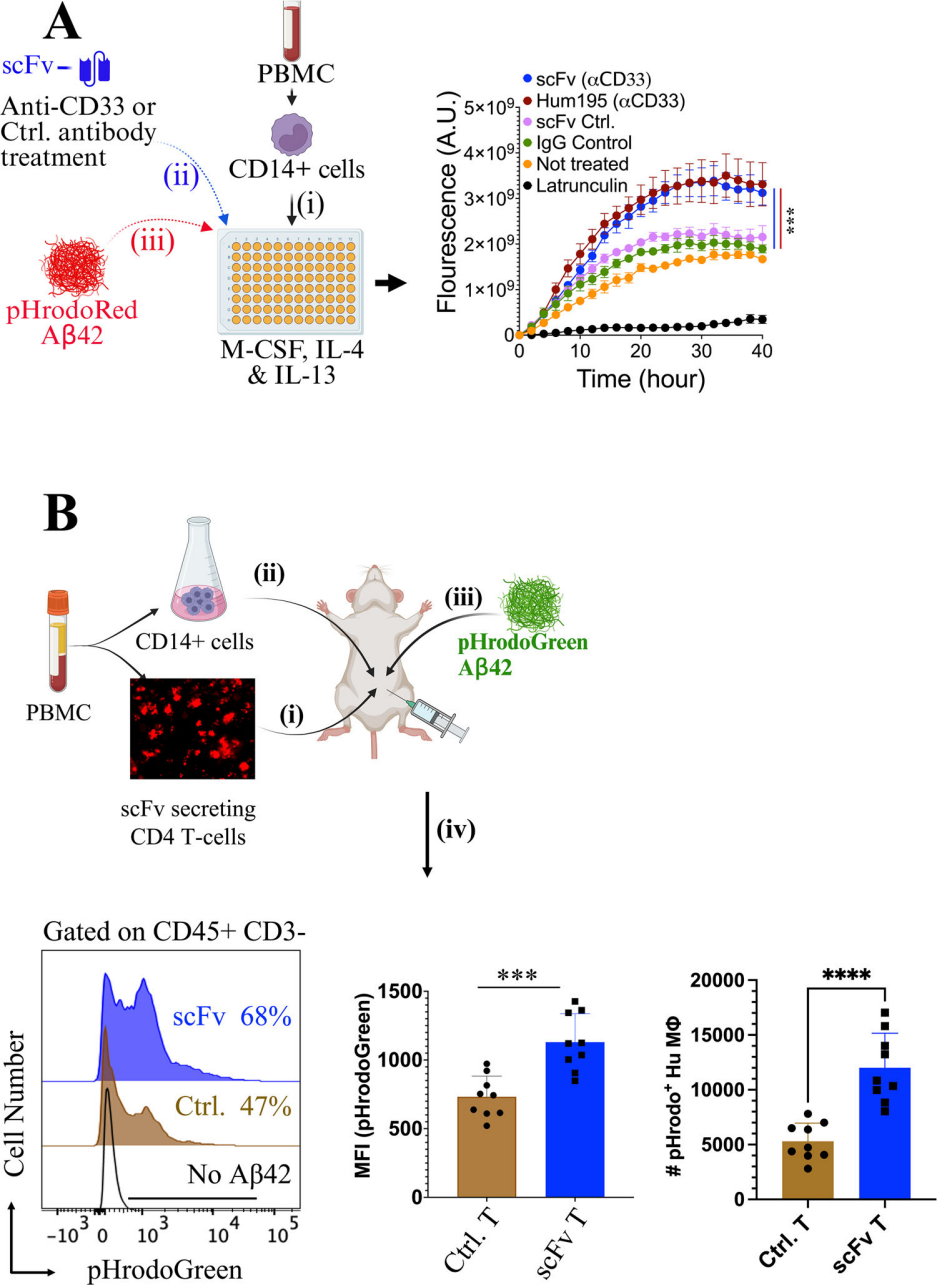
Anti-CD33 scFv treatment increased Aβ42 uptake by human macrophages and microglia. (**A**) Primary human CD14+ monocytes were differentiated into macrophages as described in the materials and methods section. Macrophages were treated with: PBS (no treatment), purified anti-CD33 scFv, positive control HuM195 (1 µg/ml), control scFv (1 µg/ml), control IgG (1 µg/ml), and Latrunculin as a negative control (1 µM) for 4 h before pHrodoRed-labeled Aβ42 (1 µM) was added to the cells to initiate phagocytosis, and the quantification of continuous live imaging of pHrodoRed-Aβ42 uptake by Hu-Mø shown in fluorescent (A.U.) was taken every 2 h for 48 h. means ± SEM; *n* = 4. (***P < 0.001) as determined by Tukey’s multiple comparisons test. (**B**) To model therapeutic conditions in vivo, mice received intraperitoneal co-injections of two million control or anti-CD33 scFv-secreting CD4 T cells together with one million primary human macrophages (Hu-Mø) and pHrodoGreen-Aβ42 (200 µg/mouse). Seventy-two hours later, cells were recovered from the peritoneal cavity and analyzed by flow cytometry. The left panel shows a representative histogram from gated human CD45 + CD3− Hu-Mø indicating the percentage of pHrodoGreen-positive cells. The middle panel quantifies the MFI within the pHrodoGreen-positive gate (per-cell uptake). The right panel reports absolute pHrodo+ Hu-Mø counts, reflecting the number of phagocytically active macrophages recovered. Data are shown as mean ± SD across three independent experiments (*n* = 9 total mice); group comparisons were by unpaired two-tailed *t* test, with significance indicated on the plots (***P < 0.001; ****P < 0.0001). Source data are available online for this figure.

Encouraged by these results, we next studied the effect of purified anti-CD33 scFv on the kinetics of pHrodo-Aβ42 uptake by primary human macrophages in real time using a Cytation 5 imaging system. Blocking CD33 on Hu-Mø by scFv or HuM195 resulted in a time-dependent increase in phagocytosis of pHrodo-Aβ42 (Fig. 2A). However, no changes in the basal pHrodo-Aβ42 phagocytosis were observed in control scFv, IgG1-treated cells, and untreated groups. Moreover, the uptake of pHrodo-Aβ42 was blocked by Latrunculin, a phagocytosis inhibitor, used as a negative control. Together, these results demonstrated that the anti-CD33 scFv can significantly enhance the phagocytosis of Aβ42 in human macrophages over time as potently as its parental antibody, HuM195.

To investigate whether CD4 T-cell-secreted scFv can bind to its target in live animals and modulate phagocytosis of macrophages in vivo, we injected either two million control or scFv-secreting CD4 T-cells into the intraperitoneal cavity of mice along with a million Hu-Mø and pHrodo-Aβ42 (200 μg) (Fig. 2B, top panel). Seventy-two hours later, cells were isolated from the peritoneal cavity, and human CD45 + CD3- cells were analyzed for pHrodo-Aβ42 signal via flow cytometry.

Similar to our in vitro findings, Aβ42 phagocytosis was enhanced in mice treated with anti-CD33 scFv-secreting CD4 T-cells compared to those treated with control CD4 T-cells (Fig. 2B, bottom panels). These results demonstrate that the CD4 T-cells secreting anti-CD33 scFv can enhance Aβ42 phagocytosis of Hu-Mø by blocking their cell surface CD33 in vivo.

### Engineering regulatory like CAR-FoxP3 CD4 T-cell as an scFv delivery vehicle into the brain

Infusion of an anti-CD33 scFv or mAb systemically is challenging for the treatment of AD due to the poor penetration into the brain and off-target effects due to the expression of CD33 on myeloid and monocytic cells. We therefore sought to genetically engineer T-cells that could efficiently enter the brain and secrete anti-CD33 scFv locally to promote the phagocytic function of microglia and brain-borne macrophages. T-cells engineered to express anti-GD2 CAR have been shown to remain localized in the brain (Richman et al, 2018). However, anti-GD2 CAR T-cells are limited by on-target toxicity due to reactivity with GD2+ neurons (Richman et al, 2018; Cook et al, 2023; Epperla et al, 2023; Velasco et al, 2023). Therefore, regulatory CD4 T-cells (CAR Tregs) engineered to express an anti-GD2 CAR would be a better choice as they are not inherently inflammatory. However, isolating a pure FoxP3+ Treg population is difficult due to the lack of specific cell surface markers and their low abundance in peripheral blood (1–5%) (Appendix Fig. S2). Therefore, we engineered polyclonal CD4 T-cells into regulatory-like CAR-FoxP3 CD4 T-cells to minimize their possible toxicity in vivo. CD4 T-cells were chosen due to their high abundance in the peripheral blood, ease of isolation due to specific cell surface markers, and reduced inflammatory properties compared to CD8 T-cells. In addition, FoxP3 was ectopically expressed to induce Treg-like phenotype thereby decreasing the risk of toxic inflammation and graft-versus-host disease (GvHD).

**Figure 3:**
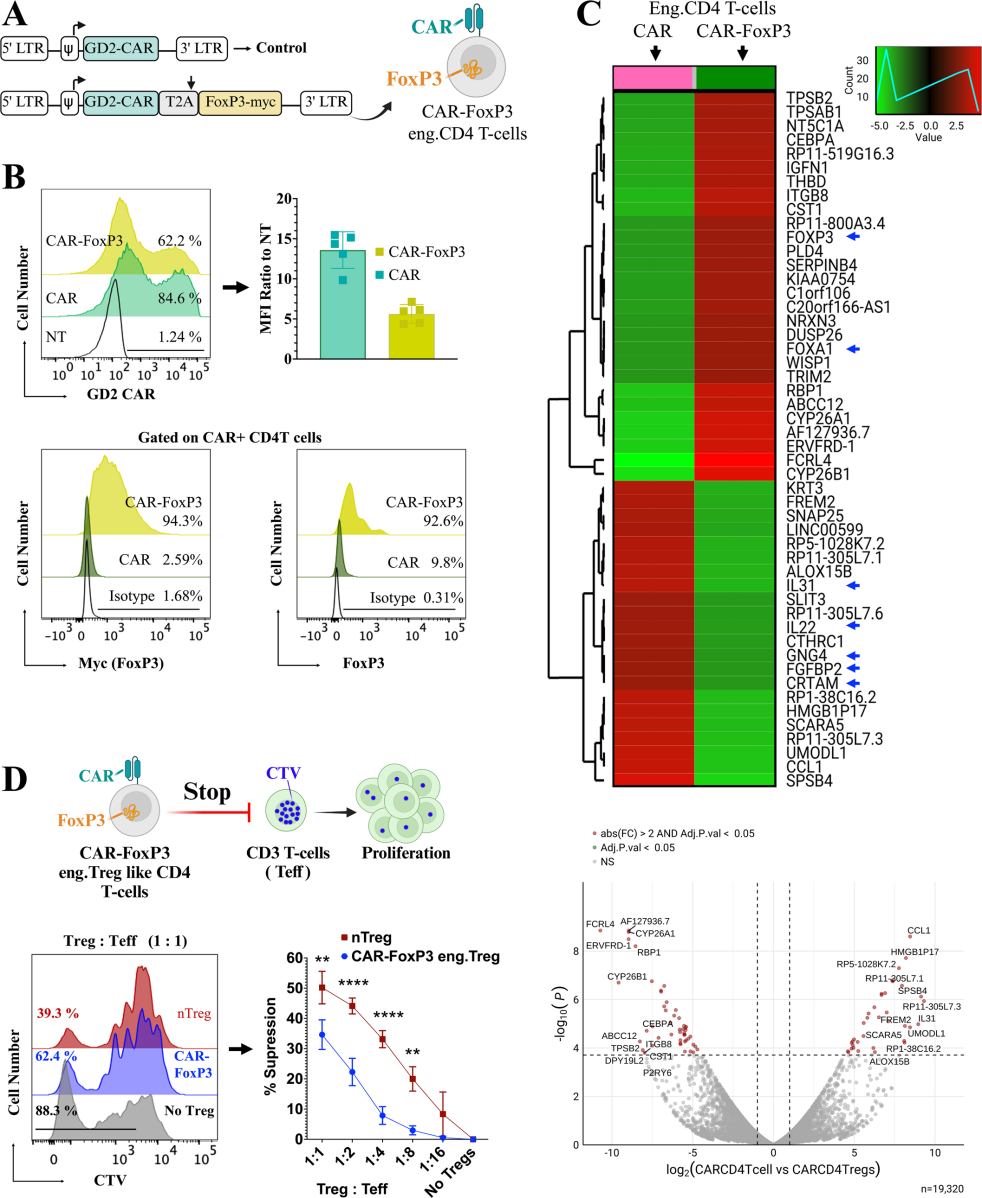
Engineering regulatory like CAR-FoxP3 CD4 T-cells. (**A**) Schematic representation of retroviral vectors. The lower vector represents the anti-GD2 CAR-FoxP3 construct in an SFG-*γ*-retroviral vector. The human FoxP3 gene was added next to the CAR using a self-cleavable T2A peptide sequence to get the final *α*GD2scFv-41BB-CD3*ζ*-T2A-FoxP3 construct between the LTR. A myc tag was added at the N-terminal of the FoxP3 to detect its expression. The upper vector that carried only the anti-GD2 CAR gene was used as a control against FoxP3 expression. (**B**) Representative overlaid histograms show the efficiency of retrovirus transduction. The top left histogram shows the percentage of CAR-positive staining on CD4 T-cells transduced with retrovirus for CAR-FoxP3 (yellow) and CAR (green). The top right graph shows MFI data of CAR-positive staining of five different transductions. The bottom left representative histogram shows intracellular flow cytometry staining of myc-tag, confirming a concurrent expression of exogenous FoxP3 in CAR-FoxP3 retrovirus transduced CD4 T-cells (yellow) but not in CAR transduced CD4 T-cells (Green). The bottom right shows a direct FoxP3 staining that also demonstrates high expression of FoxP3 protein in CAR-FoxP3 transduced CD4 T-cells compared with CAR CD4 T-cells alone. (**C**) A representative heatmap (top) and volcano plots (bottom) of differentially expressed genes. The heatmap shows the top 50 differentially expressed genes in CAR-FoxP3 eng.CD4 T-cells versus CAR eng.CD4 T-cells. The blue arrow highlights the genes that are known to be associated with inflammation or regulatory function. The volcano plot presents the nominal *P* value against fold-change after log transformation for every gene represented by each dot. Red dots indicate differentially expressed genes with *P* value < 0.05; gray dots indicate non-significant (NS) genes. (**D**) CAR-FoxP3 eng.CD4 T-cells vs nTreg mediated immunosuppression assay. CellTrace Violet (CTV) dye-labeled CD3 effector T-cells (Teff) were cultured alone or mixed at different ratios with either nTregs or CAR-FoxP3 eng.CD4 T-cells, and activated with anti-CD3 antibody. After 72 h, the suppressive function of Tregs was determined by assessing the inhibition of CTV dilution by flow cytometry analysis. The bottom left shows a representative histogram of Treg-mediated immunosuppression at a 1:1 ratio. The solid line in the histogram represents the gating window of the baseline percent proliferation of Teff cells without any Tregs. The bottom right graph shows the suppression of the proliferation of Teff mediated by CAR-FoxP3 eng.CD4 T-cells versus nTregs at different Treg: Teff ratios. Graphs show the mean ± SD of three pooled independent experiments performed in triplicate. Statistical significances were calculated using two-way ANOVA with Mixed-effects analysis comparing each cell mean with the other cell mean in that row (**P < 0.01; ****P < 0.0001).

To engineer regulatory-like CAR-FoxP3 CD4 T-cells, we constructed a retrovirus plasmid including an affinity-enhanced anti-GD2-E101K CAR and an exogenous myc-tagged FoxP3 gene separated by a T2A cleavable sequence (Fig. 3A). A GD2 CAR-only expressing retrovirus construct was used as a negative control for cells engineered with exogenous FoxP3. Retroviral transduction efficiency of CD4 T-cells was determined by flow cytometry using an antibody against anti-GD2 CAR.

**Figure EV2:**
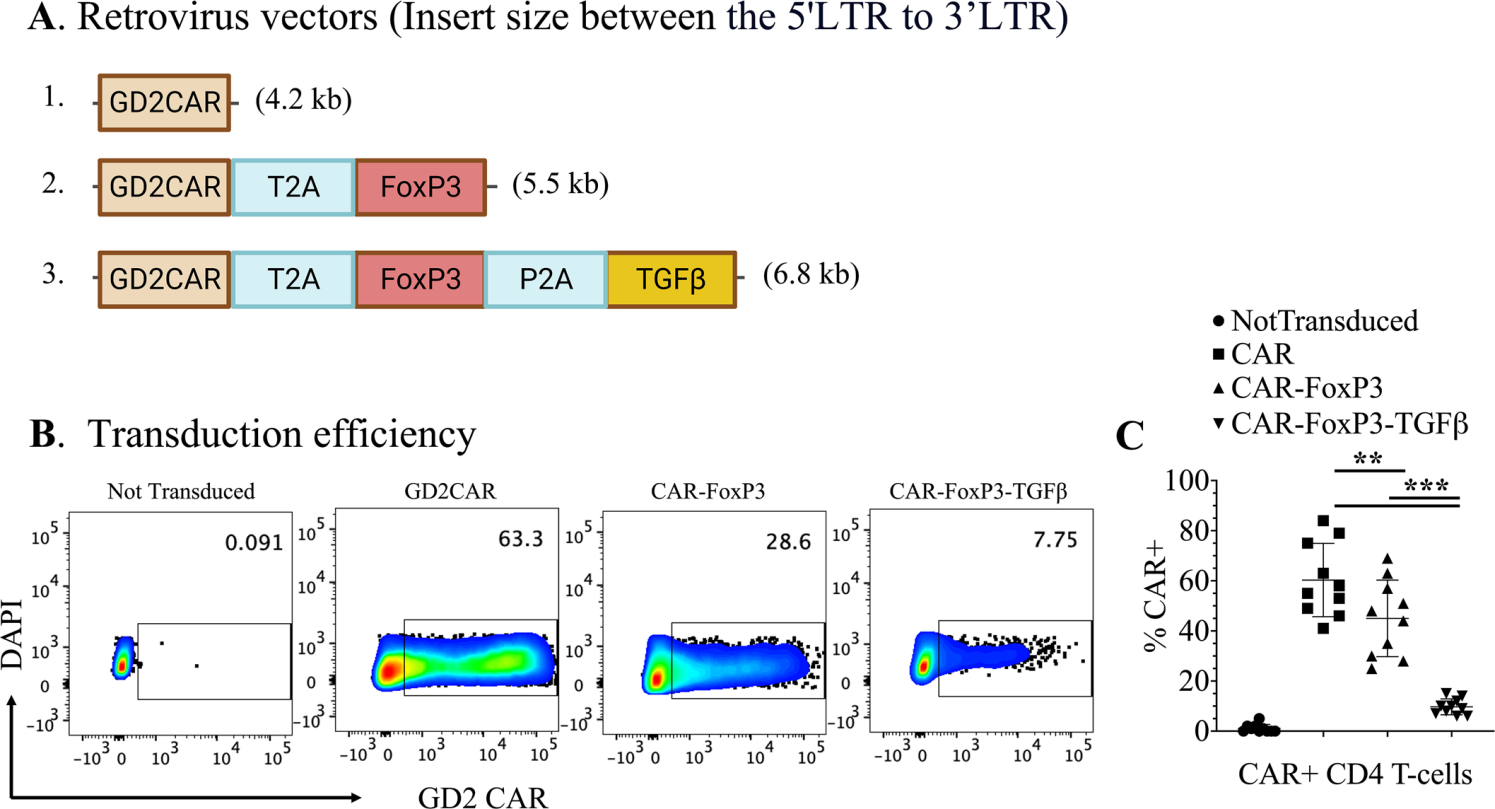
Transduction efficiency of mono-cistronic, bi-cistronic, and tri-cistronic retroviral constructs. (**A**) SFG retrovirus vectors were constructed expressing: 1. CAR alone (mono-cistronic), 2. CAR-FoxP3 (bi-cistronic), 3. CAR-FoxP3-TGFβ (tri-cistronic) between their 5’LTR and 3’LTR regions. (**B**) Representative dot plots show transduction efficiency of respective retroviral vectors as percent CAR-positive CD4 T-cells on D7 post-transduction. (**C**) Showing percent transduction mean with SD of ten experiments. The Two-way ANOVA multiple comparisons statistical analysis was performed using Prism 10 (**P < 0.01; ***P < 0.001).

We used CAR-positive CD4 T-cells transduced above 40% for in vitro and in vivo studies. Transduction efficiency of up to 76% was obtained with the CAR-FoxP3 bi-cistronic retrovirus. CD4 T-cells, GD2 CAR-FoxP3 cells, and GD2 CAR T cells were transduced with 62% and 84% efficiency, respectively (Fig. 3B, top row). Intracellular flow cytometry analysis of myc-tag confirmed the concurrent expression of exogenous FoxP3 in CAR-positive CD4 T-cells (Fig. 3B, lower left). Direct FoxP3 staining also confirmed high expression of FoxP3 protein in CAR-FoxP3 engineered CD4 T-cells compared to control CAR CD4 T-cells alone (Fig. 3B, lower right). These results demonstrated that these transduced CD4 T-cells expressed both a CAR on the cell surface and intracellular exogenous FoxP3 protein. Tri-cistronic retrovirus vectors and other designs produced inefficient transduction and were not pursued further (Fig. EV2). Therefore, we decided to use the bi-cistronic CAR-FoxP3 construct for the entirety of our study.

**Figure EV3:**
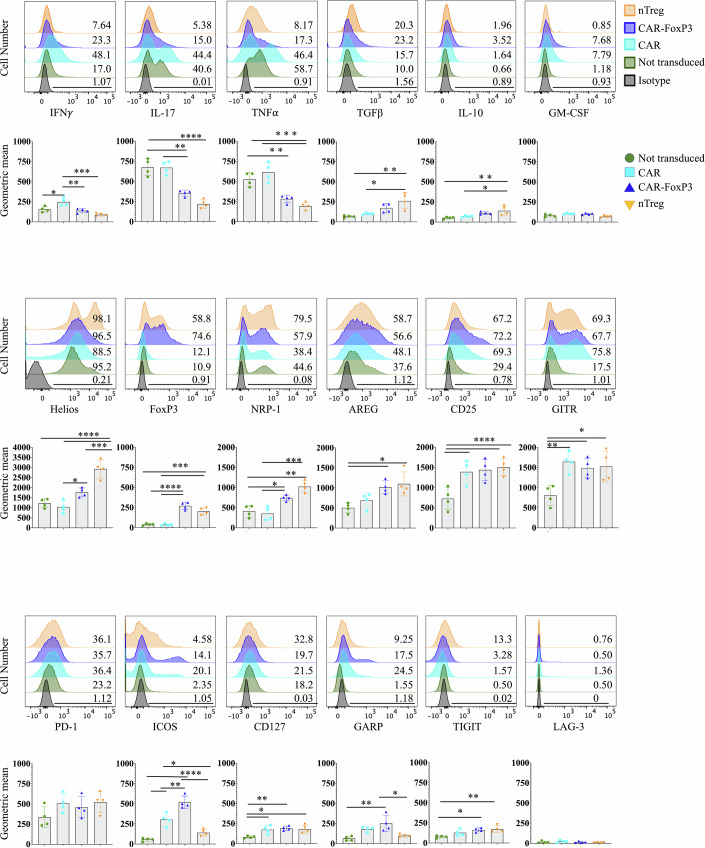
Intracellular and cell surface protein characterization. Representative overlaid histograms showing the expression of intracellular and cell surface markers among the indicated T-cells. Six days post-transduction, flow cytometry-sorted CAR-positive CD4 T-cells (CAR and CAR-FoxP3), untransduced CD4 T-cells, and nTreg were restimulated with anti-CD3 antibodies and cultured in media supplemented with TGFβ, all-trans retinoic acid, and IL-2. Seventy-two hours post-stimulation, cells were stained for the selected cell surface markers. For intracellular staining, on day 3 post-restimulation, cells were incubated with the protein transport inhibitor GolgiStop for four hours before fixation/permeabilization and intracellular staining and analyzed by flow cytometry. The bar diagram below each histogram shows the geometric mean with SD of the respective staining from four independent stainings. The two-way ANOVA multiple comparisons statistical analysis was performed using Prism 10 (*P < 0.05; **P < 0.01; ***P < 0.001).

To make CAR-FoxP3 engineered CD4 T-cells adopt regulatory T-cell-like phenotypes, we sorted CAR-positive cells via flow and expanded them using anti-CD3 mAb restimulation (1 µg/ml) in complete RPMI media supplemented with 10 ng/ml TGFβ, 10 nM all-trans retinoic acid, and 500 IU IL-2 (Schmidt et al, 2016). Cell surface and intracellular cytokine characterizations of these cells showed that while there was a significant downregulation of inflammatory cytokines such as IFN*γ*, IL-17, and TNF*α*, expression of genes like Amphiregulin (AREG), Neuropilin-1 (Nrp-1), ICOS, TGFβ, and Helios was upregulated in CAR-FoxP3 CD4 T-cells compared to both CAR alone and untransduced CD4 T-cells (Fig. EV3). TGFβ is critical in maintaining the self-tolerance and immunosuppressive functions of Tregs (Wan and Flavell, 2007). Amphiregulin and Helios are known to be associated with nTreg functions (Zaiss et al, 2013; Kim et al, 2015). We observed high cell surface protein Nrp-1 expression in nTreg and also in our CAR-FoxP3 engineered CD4 T-cells. Nrp-1 is a transmembrane glycoprotein known to be upregulated in TGFβ-induced Treg and has an immunosuppressive function (Chen et al, 2022). Increased expression of other cell surface proteins, such as CD25, PD-1, and GITR was also detected; however, their expression was not significantly different from the other groups (Fig. EV3).

RNAseq analysis of the top 50 differentially expressed genes also confirmed the downregulation of inflammatory genes such as IL-31 (Borgia et al, 2022), IL-22 (Keir et al, 2020; Lindahl and Olsson, 2021), GNG4 (Duan et al, 2022), FGFBP2 (Nicolet et al, 2021; Haskamp et al, 2022), and CRTAM (Takeuchi et al, 2016) and upregulation of regulatory genes, FOXA1 (Liu et al, 2014) and FoxP3 (Rudensky, 2011)in CAR-FoxP3 eng.CD4 T cells (Fig. 3C). This alteration in gene expression confirmed that exogenous FoxP3 expression reduced the inflammatory gene expression in the CAR-FoxP3 eng.CD4 T-cells and drove them towards regulatory-like T-cells.

Next, we performed an in vitro suppression assay to evaluate whether CAR-FoxP3 eng.CD4 T-cells have functionally adopted a regulatory T cell-like suppressive phenotype. The CAR-FoxP3 eng.CD4 T-cells or nTreg were mixed at different Treg:Teff ratios with cell trace violet (CTV) dye-labeled CD3 effector T-cells as (Teff) and stimulated with anti-CD3 antibody. Seventy-two hours later, suppression of proliferation of the CD3 Teff cells was analyzed by flow cytometry. We observed suppression of proliferation of the CD3 Teff cells by both nTreg and CAR-FoxP3 eng.CD4 T-cells in a distinctive Treg: Teff ratio-dependent manner. Around 50% of suppression of proliferation of Teff was observed with nTreg at a 1:1 ratio, and a linear trend was observed at various dilutions until 1:16 nTreg: Teff. A similar pattern of suppression of Teff cells was observed by our CAR-FoxP3 eng.CD4 T cells, though of less magnitude, confirming a phenotypic change in their effector behavior (Fig. 3D).

### CAR-FoxP3-engineered Treg-like CD4 T-cells can migrate into and persist in the brain and are nontoxic

**Figure 4:**
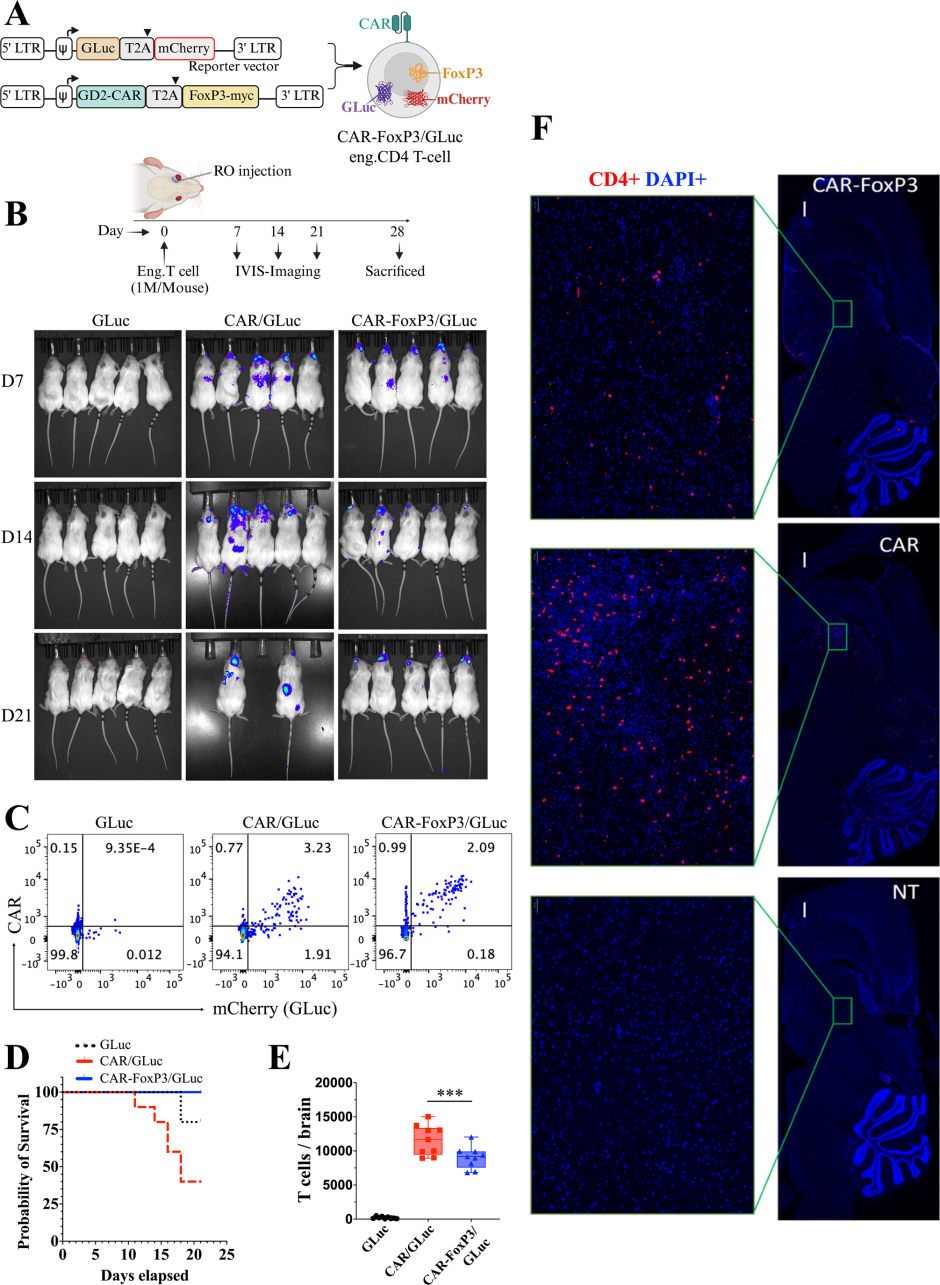
CAR-FoxP3 eng.CD4 T-cells are retained in the brain. (**A**) Schematic retroviral vectors. The upper vector represents the Gaussia luciferase (GLuc) reporter system for in vivo imaging. An mCherry gene was inserted next to GLuc using a T2A linker system as a marker of transduction. The lower retrovirus vector is an anti-GD2 CAR-FoxP3 construct. The retrovirus with the anti-GD2 CAR gene alone was used as a control against exogenous FoxP3 expression (not shown in the above scheme). Also, see Appendix Fig. S3. (**B**) BLI images: three million double-transduced CAR-FoxP3/GLuc CD4 T cells, and as controls CAR/Gluc CD3 T cells, or GLuc-alone eng.CD3 T-cells were injected per mouse intravenously (RO=Retroorbital). In vivo, localization of the injected cells was followed using the GLuc substrate Coelenterazine via an IVIS imaging system. IVIS-BLI images are representative of three independent experiments (*n* = 15 for each group) and show signals from different time points in the brain and periphery. (**C**) A representative flow cytometry analysis of the brain lysate preparation shows the presence of mCherry and CAR double-positive T-cells in both CAR+ groups on day 21. Cells were gated on DAPI-negative and human CD45+ populations. (**D**) Kaplan–Meier survival analysis showing significant differences in mortality induced by CAR-FoxP3/Gluc CD4 T cells, and CAR/Gluc or GLuc alone eng.CD3 T-cells from three independent experiments (*n* = 15 for each group). Log-rank (Mantel–Cox) test *χ*^2^ = 8.177. *P = 0.0168. (**E**) Box-and-whisker plot showing the total number of accumulated engineered T-cells in the mouse brain on day 21 from the respective groups. Data are from three independent experiments, *n* = 9. The center line represents the median. The box bounds indicate the 25th and 75th percentiles. Whiskers extend to the minimum and maximum observed values. Individual symbols represent biological replicates. The two-way ANOVA multiple comparisons statistical analysis was performed using Prism 10 (***P < 0.001, ****P < 0.0001). To shorten the names, we used the abbreviation GLuc instead of GLuc-mCherry; however, GLuc is expressed together with mCherry. (**F**) Representative immunofluorescence staining of the brain sections confirmed the presence of human CD4 T-cells in both CAR-FoxP3 eng.CD4 T-cells and CAR eng.CD3 T-cells groups but not in the non-transduced (NT) group on day 14 in a parallel experiment (*n* = 3 for each group). The inset highlights brain areas proximal to the midbrain/hypothalamus. CD4 T-cells that were retained in the hippocampus are shown in Fig. EV4. Scale bars, 50 μm.

Affinity-enhanced anti-GD2 CAR-expressing CD3 T-cells have previously been reported to be highly neurotoxic (Richman et al, 2018; Cook et al, 2023; Epperla et al, 2023; Velasco et al, 2023). Hence, here we demonstrate that CD4 T-cells engineered to express anti-GD2 CAR and FoxP3 are relatively less toxic and equally efficient in migrating into and persisting in the CNS in immunodeficient mice. We utilize the Gaussia luciferase (GLuc) reporter system to track cell localization in vivo by Bioluminescence imaging (BLI). CD4 T-cells were double transduced with two retroviruses to deliver CAR-FoxP3 and GLuc-mCherry genes. mCherry gene was included in the GLuc vector as a marker of transduction (Fig. 4A). CD3 T-cells double transduced with CAR (without FoxP3) and GLuc retroviruses were used as a control for exogenous FoxP3-engineered cells. Gluc-alone transduced CD3 T-cells were used as a control for CAR expression. Double transduction of T-cells was confirmed by cell-surface CAR staining and intracellular mCherry expression (Appendix Fig. S3).

For biodistribution studies in vivo, three million double transduced CAR-FoxP3/GLuc eng.CD4 T- cells, CAR/GLuc eng.CD3 T-cells, or just GLuc eng.CD3 T-cells were injected intravenously per mouse in three groups. The mice were imaged weekly by the IVIS-BLI system to track the localization of the injected cells using GLuc substrate, 500 µg of water-soluble coelenterazine (Fig. 4B). By day 7 post-injection, we detected BLI signals in the brains of mice in both CAR-FoxP3/GLuc eng.CD4 T-cells and CAR/GLuc eng.CD3 T-cells. The signal in CAR-FoxP3/GLuc eng.CD4 T-cells gradually increased over the weeks in the brain, and no death was seen in this group up to day 21. In contrast, peripheral and CNS-specific signals for the CAR/GLuc eng.CD3 T-cells rapidly increased by day 14. As a consequence of activation of these accumulated T cells, mice in this group began to die by the 3rd week (day 21) (Fig. 4B). However, we did not observe BLI signals in the GLuc alone transduced CD3 T-cell group, and no mortality was seen either, indicating that GD2 CAR expression is needed for their retention in the brain. Mice were sacrificed on day 21 with CO_2_ and perfused with PBS, and their brain homogenates were prepared for flow cytometry to detect engineered T-cells that accumulated in the brain. Flow cytometry analysis confirmed mCherry (GLuc) and CAR double-positive cells in the brain homogenates of both CAR-FoxP3/GLuc and CAR/GLuc groups, confirming their retention in the brain (Fig. 4C). However, we did not detect CAR-negative GLuc alone eng. T-cells in the brain. These findings suggest that a brain antigen-reactive CAR expression is required for the engineered T-cells to be retained in the brain; additionally, FoxP3 expression is needed for these cells to remain nontoxic, as mice injected with CAR/GLuc eng. CD3 T-cells had poorer survival than those that received CAR-FoxP3/GLuc eng.CD4 T-cells (Fig. 4D). However, the total number of CAR-FoxP3/GLuc CD4 T-cells found in the brain was significantly lower than CAR/GLuc eng.CD3 T-cells (Fig. 4E). In parallel experiments, immunofluorescence (IF) staining of mouse brain sections with anti-human CD4 and CD3 antibodies confirmed the presence of engineered T-cells in both groups but not in the non-transduced (NT) T-cell group (Fig. 4F).

### Human IL-2 expression makes anti-CD33 scFv/CAR-FoxP3 eng.CD4 T-cells survive longer

**Figure 5:**
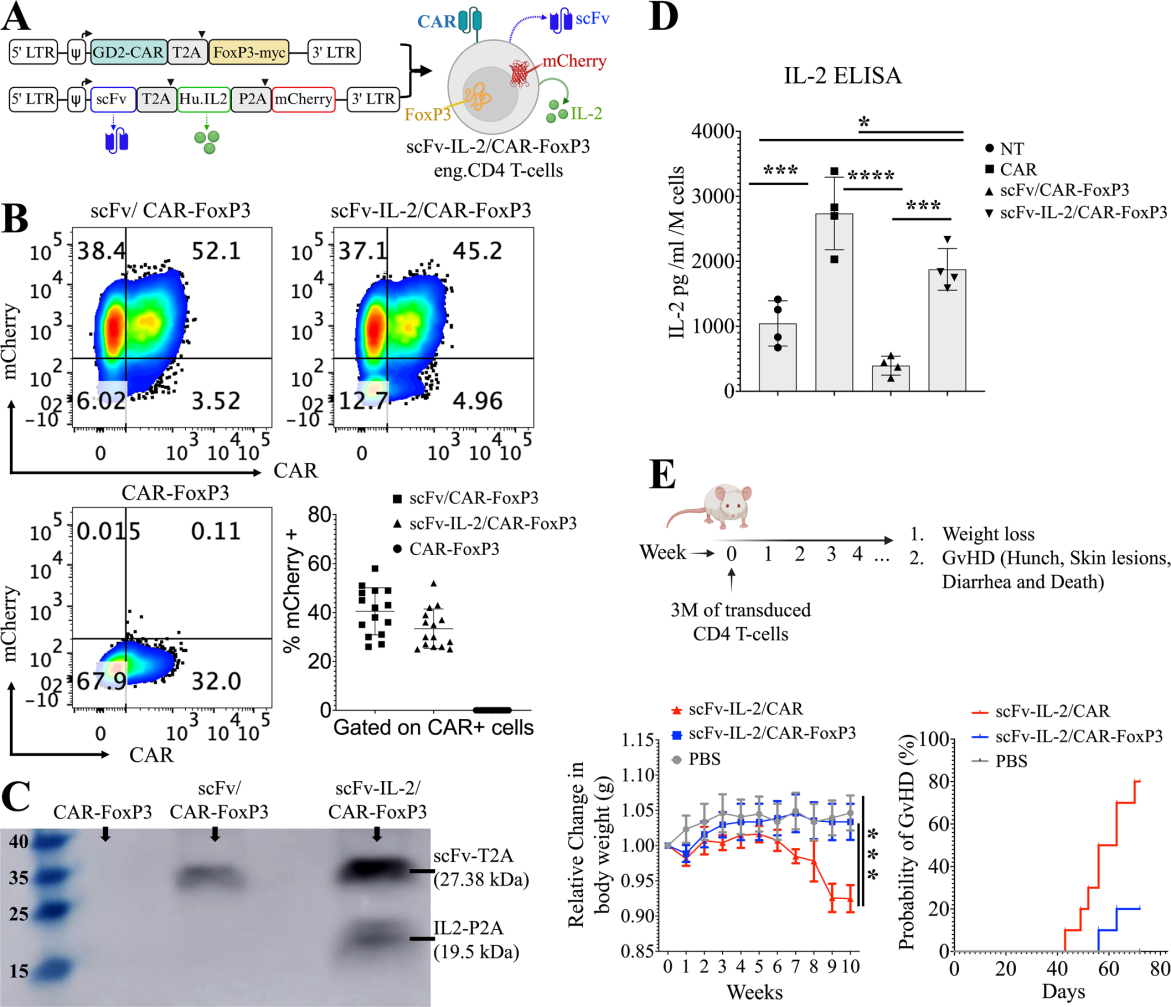
Human IL-2 expression makes anti-CD33 scFv/CAR-FoxP3 eng.CD4 T-cells survive better in vivo. (**A**) Schematic retroviral vectors. The upper vector is the CAR-FoxP3 construct. The lower vector shows IL-2 inserted scFv construct in the order of scFv-T2A-IL-2-P2A-mCherry. The retrovirus vector with scFv-T2A-mCherry was only used as a control for exogenous IL-2 expression (not shown in the above scheme). (**B**) Representative flow cytometry dot plots showing double transduction efficiency for scFv/CAR-FoxP3 (upper left) and scFv-IL-2/CAR-FoxP3 (upper right) retroviruses. The lower left panel is representative of a single retrovirus transduction for CAR-FoxP3 as a control for mCherry. The lower right bar graph with individual data points shows the percentage mean +/− SD of double transduced CD4 T-cells used for in vitro and in vivo experiments. The percentage of mCherry-positive cells shown is from CAR + CD4 T-cells. (**C**) Western blot using an anti-2A peptide antibody showing eng.CD4 T-cells secreted IL-2 and scFv-specific bands. (**D**) ELISA detection of cell-secreted IL-2 in the supernatant of the eng.CD4 T-cells. Individual data points are shown with mean +/− SD. The two-way ANOVA multiple comparisons statistical analysis was performed using Prism 10 (*P < 0.01, ***P < 0.001, ****P < 0.0001). (**E**) In vivo studies in which three million respective eng.CD4 T-cells were injected intravenously, and mice were monitored for 10 weeks for related toxicity, such as weight change and GvHD (see inset scheme). The lower left graph shows the change in body weight among different groups over 10 weeks after respective eng.CD4 T-cells were injected; the lower right graph shows percent mice with GvHD among these treatment groups. Data are from three independent experiments. The two-way ANOVA multiple comparisons statistical analysis was performed for the relative change in body weight using Prism 10. means ± SEM; *n* = 10 for each group (***P < 0.001). Kaplan–Meier curve showing significant difference in percentage probability of GvHD occurrence induced by PBS, scFv-IL-2/CAR, or scFv-IL-2/CAR-FoxP3 eng.CD4 T-cells from three independent experiments. *n* = 10 for each group. Log-rank (Mantel–Cox) test *χ*^2^ = 18.10. ****P = 0001. Source data are available online for this figure.

Although we detected a successful retention of CAR-FoxP3 eng.CD4 T-cells in the CNS of mice, the total T cell number was significantly lower in the brain than that of CAR eng.CD3 T-cells (Fig. 4E). This could be due to the high expression of exogenous FoxP3, which is known to inhibit the production of IL-2 (Wang et al, 2018); consequently, FoxP3 could weaken their overall health and survival. nTregs depend highly on other T-cells to provide them with IL-2 for their function in vivo (Harris et al, 2023). However, no IL-2-producing cells are naturally present in the immunodeficient mice; hence, the long-term survival of our CAR-FoxP3 eng.CD4 T-cells could be compromised in vivo. To enhance the survival in vivo, we decided to remodel CAR-FoxP3 eng.CD4 T-cells such that they secrete autocrine human IL-2 along with anti-CD33 scFv. We inserted a human IL-2 gene in the scFv-mCherry vector using P2A peptide linker sequences to get the final construct as scFv-T2A-IL2-P2A-mCherry (abbreviated as scFv-IL-2). We sequentially transduced CD4 T-cells with CAR-FoxP3 and scFv-IL-2 retroviruses to get the final scFv-IL-2/CAR-FoxP3 eng.CD4 T-cells that make cell surface CAR, intracellular FoxP3, mCherry, and secrete anti-CD33 scFv and IL-2 (Fig. 5A). As the retroviral construct is transcribed under the viral LTR promoter, exogenous IL-2 expression likely will not be inhibited by the FoxP3 expression nor activated by the CAR.

CAR surface staining and intracellular mCherry expression confirmed the double retrovirus transduction (Fig. 5B). The mean double transduction efficiency of scFv-IL-2/CAR-FoxP3 retroviruses was around 30 percent, slightly lower than that of scFv/CAR-FoxP3 retroviruses, which was about 40 percent (Fig. 5B). For studies in vivo, we enriched the scFv-IL-2/CAR-FoxP3 eng.CD4 T-cells were sorted via flow cytometry when the double transduction efficiency was less than 40 percent. A western blot confirmed the secreted scFv and IL-2 in the supernatant of double-transduced scFv-IL-2/CAR-FoxP3 eng.CD4 T-cells using an anti-2A peptide-specific antibody. The anti-2A peptide antibody cross-reacts with both T2A and P2A peptides. Therefore, we detected both an IL2-P2A-specific band of around 19.5 kDa and an scFv-T2A-specific band of 27 kDa (Fig. 5C). However, the supernatant from CD4 T-cells double transduced with scFv/CAR-FoxP3 retroviruses showed no IL-2-specific band in the western blot, confirming a successful secretion of exogenous IL-2 and anti-CD33 scFv by scFv-IL-2/CAR-FoxP3 eng.CD4 T-cells. ELISA detection confirmed reduced IL-2 production in our CAR-FoxP3 eng.CD4 T cells compared to both untransduced and CAR only CD4 T-cells (Fig. 5D). On the other hand, scFv-IL-2/CAR-FoxP3 eng.CD4 T-cells produced two-fold higher IL-2 in the supernatant compared to non-transduced CD4 T-cells (Fig. 5D).

**Figure EV5:**
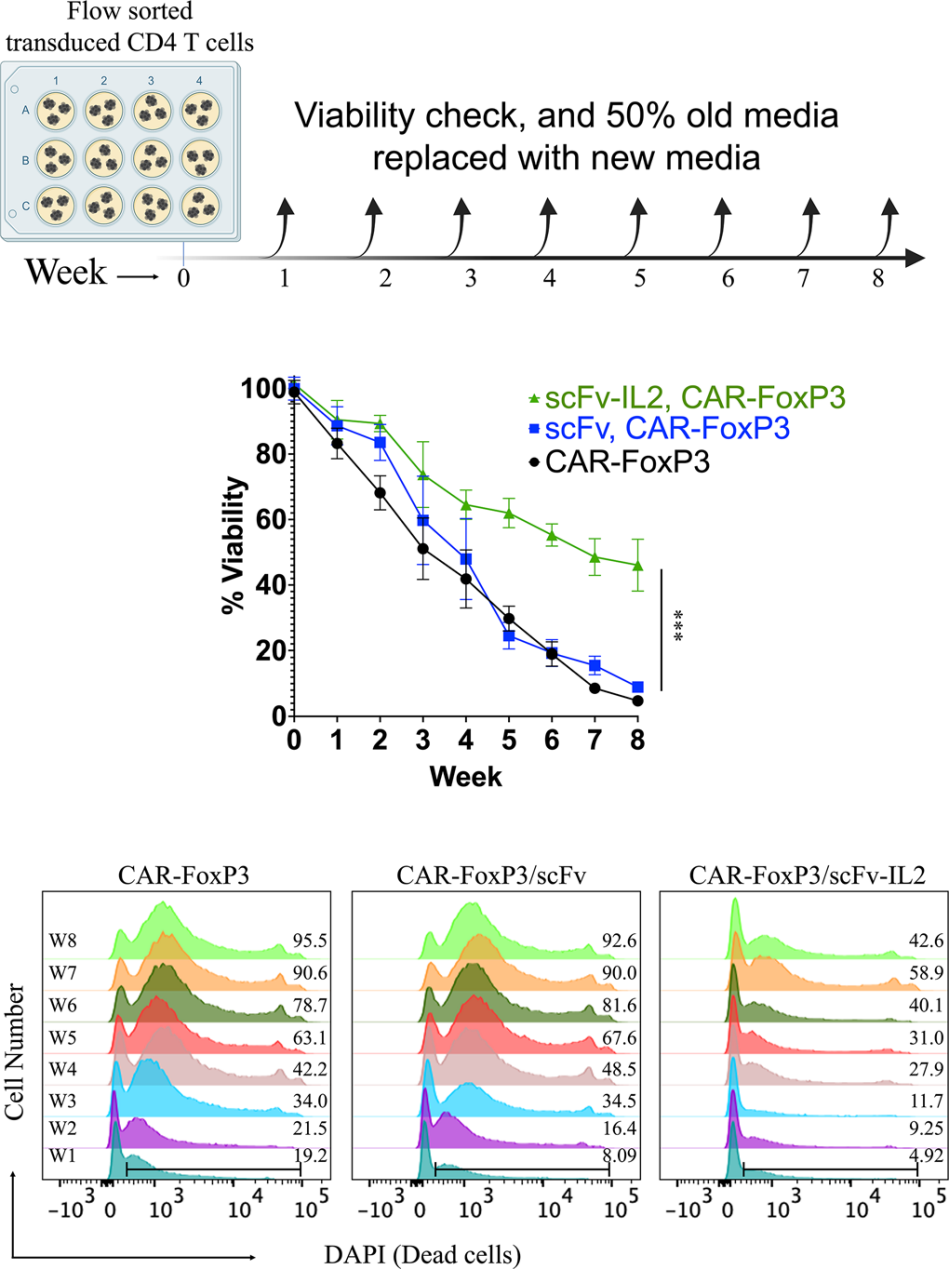
In vitro long-term survival of eng. CD4 T-cells in IL-2-free culture media. Human primary CD4 T-cells were double transduced to express scFv-IL-2/CAR-FoxP3, scFv/CAR-FoxP3, or CAR-FoxP3 alone. The cells were then sorted using anti-GD2 CAR staining and mCherry expression, and cultured for 8 weeks in IL-2-free RPMI complete media at 1 million /ml /well in 12-well plates. The viability of the cells was analyzed weekly by flow cytometry using DAPI staining. Also, 50% of the cultured media was replaced with new RPMI media weekly (scheme). The graph in the middle shows the percent viability of the three groups over eight weeks. The bottom is a representative overlaid histogram showing each group’s DAPI-positive staining from week 1 to week 8. The two-way ANOVA multiple comparisons statistical analysis was performed using Prism 10. means ± SD; *n* = 3. (***P < 0.001).

These results confirmed that the expression of the exogenous IL-2 gene was not inhibited in the scfv-IL-2/CAR-FoxP3 eng.CD4 T-cells. The long-term survival in vitro of scFv-IL-2/CAR-FoxP3 eng.CD4 T-cells were also much better, as their viability remained above 40 percent even after 8 weeks of culture in IL2-free media. This compared favorably to both scFv/CAR-FoxP3 and CAR-FoxP3 eng.CD4 T-cells (Fig. EV5).

The long-term in vivo persistence and possible consequential toxicity, such as weight loss and GvHD, were compared among scFv-IL-2/CAR-FoxP3, scFv-IL-2/CAR eng.CD4 T-cells, and PBS-treated groups. Eng.CD4 T-cells were injected intravenously, and associated toxicity, such as body weight loss in addition to any positive signs of GVHD (hunched posture, skin lesions, diarrhea, and death), was monitored over 10 weeks. The absence of exogenous FoxP3 expression in scFv-IL-2/CAR eng.CD4 T-cells makes them inflammatory in vivo as we observed a significant decrease in the body weight of this group (up to 20 percent drop) compared to the groups that either received scFv-IL-2/CAR-FoxP3 eng.CD4 T-cells or PBS (Fig. 5E, lower left). An increased onset of GvHD was also observed in the scFv-IL-2/CAR groups but not in the scFv-IL-2/CAR-FoxP3 group (Fig. 5E, lower right). These findings confirm that exogenous FoxP3 expression mitigates CAR-T cell–induced inflammation—whether driven by IL-2, GVHD, or both—supporting its necessity for the safer, long-term use of these cells for targeted drug delivery to the brain.

### Anti-CD33 scFv delivery into the CNS by the scFv-IL-2/CAR-FoxP3 eng.CD4 T-cells

**Figure 6:**
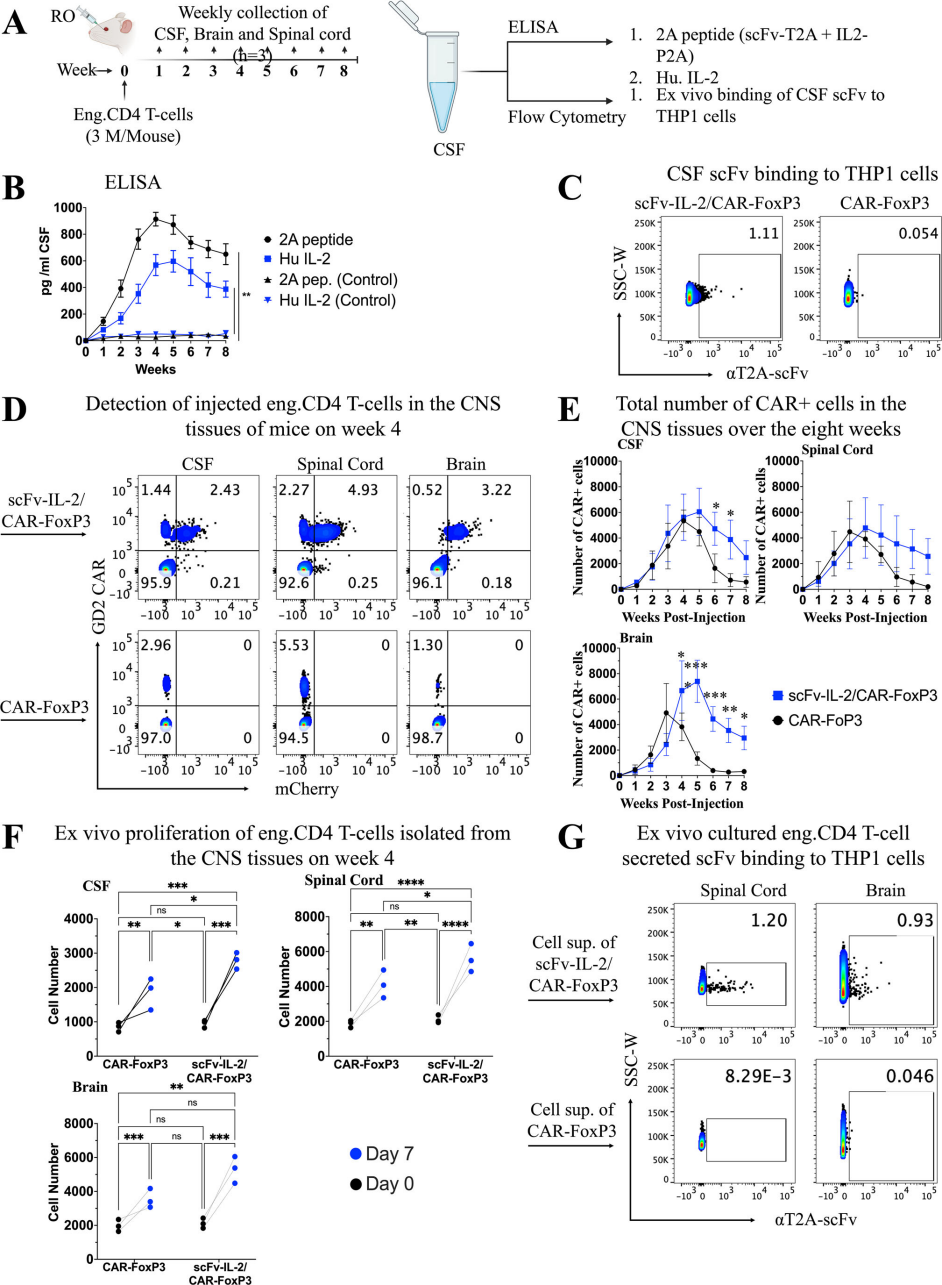
CNS delivery of scFv by the scFv-IL-2/CAR-FoxP3 eng.CD4 T-cells. (**A**) Schematic showing experimental design. Three million scFv-IL-2/CAR-FoxP3 eng.CD4 T-cells or control CAR-FoxP3 eng.CD4 T-cells were injected intravenously (RO=Retroorbital), followed by a weekly collection of CSF, spinal cord, and brain from at least three mice for up to 8 weeks. CSF samples were utilized to detect CNS-produced scFv and IL-2 using ELISA and flow cytometry. Spinal cord and brain samples were analyzed for accumulated eng.CD4 T-cells in the CNS. Data from two independent experiments are presented below. *n* = 3 for each time point/group. (**B**) ELISA detects CNS-secreted scFv and IL-2 in the CSF samples of the two groups at different time points using an anti-2A peptide (black line) and anti-human IL-2 (blue line) antibodies. Data plotted as mean +/− SD, *n* = 3 for each time point/group. The nonparametric paired *t* tests analysis was performed using Prism 10 (**P = 0.0078 for both IL-2 and T2 peptide). (**C**) Representative flow cytometry dot plots show the detection of scFv in the CSF by measuring their binding to THP1 cells. THP1-bound scFv was detected using an APC-conjugated anti-2A peptide antibody. (**D**) Representative flow cytometry dot plot showing accumulated mCherry/CAR double-positive CD4 T-cells in the CSF, spinal cord, and brain for two CAR cell types at week 4 post-injection in both groups. The mCherry is expressed with scFv-IL-2 (see Fig. 5A). (**E**) Total number of live CAR + CD4 T-cells in the CNS tissues over the 8 weeks. *n* = 3 for each time point plotted as mean +/− SD. Statistical significance determined by two-way ANOVA multiple comparisons test (*P < 0.05; **P < 0.01; ***P < 0.001; ****P < 0.0001). (**F**) Ex vivo proliferation of CNS-retained eng.CD4 T-cells that were isolated by flow sorting 4 weeks post-injection. *N* = 3 for each time point plotted individually. D0 is the day of isolation, and D7 is the day post ex vivo restimulation. Statistical significance determined by two-way ANOVA multiple comparisons test (*P < 0.05; **P < 0.01; ***P < 0.001; ****P < 0.0001). (**G**) Representative flow cytometry data show that supernatant fluid of day 7 ex vivo culture from panel F binds to THP1 cells, confirming scFv-IL-2/rCAR-FoxP3 eng.CD4 T-cells can secrete scFv upon restimulation.

Next, we examined the delivery of anti-CD33 scFv into the CNS. For that, we injected three million scFv-IL-2/CAR-FoxP3 eng.CD4 T-cells or CAR-FoxP3 eng.CD4 T-cells as a control. The cerebrospinal fluid (CSF), spinal cord, and brain were collected weekly from at least three mice from each group (Fig. 6A). ELISA results, using an anti-2A peptide antibody (detecting both secreted scFv and IL-2) in the CSF samples, confirmed the presence of cell-secreted scFv and IL-2 in the CSF of the scFv-IL-2/CAR-FoxP3 eng.CD4 T-cell treated group (Fig. 6B). This was not observed in the CAR-FoxP3 eng.CD4 T-cell-treated control group. The concentration of secreted peptides showed a rapid increase up to week 5 post-injection, with a maximum concentration of around 1000 pg/ml detected around weeks 4 and 5. This was followed by a gradual decline up to the end of the experiment at 8 weeks (Fig. 6B). An anti-human IL-2 specific antibody in a separate ELISA allowed us to differentiate between the two secreted peptides. The trend of IL-2 secretion in the CSF was similar to the anti-2A peptide ELISA, as the highest concentration of around 600 pg/ml was detected in weeks 4 and 5. These results confirmed that the anti-2A peptide concentration represented both secreted scFv and IL-2 as a cumulative value in the CSF (Fig. 6B).

Next, we assessed the ex vivo binding ability of CSF scFv to its cognate receptor, CD33, on THP1 cells. Flow cytometry analysis confirmed the binding of the CSF-derived scFv on THP1 cells, further confirming its presence in the CSF of the scFv-IL-2/CAR-FoxP3 treated group, but not in the CAR-FoxP3 control group (Fig. 6C). In addition, we also detected the presence of the scFv-IL-2/CAR-FoxP3 eng.CD4 T-cells in the CSF as mCherry/CAR double-positive cells, confirming their CSF circulation (Fig. 6D,E). In addition, accumulated scFv-IL-2/CAR-FoxP3 eng.CD4 T-cells in the spinal cord and brain tissues were also detected by flow cytometry up to 8 weeks post-injection (Fig. 6D,E). The number of the control CAR-FoxP3 eng.CD4T-cells began to decline from week 4 onwards in both tissues, confirming that IL-2 signaling is needed for CAR-FoxP3 eng.CD4 T-cells’ long-term survival in vivo (Fig. 6E).

Finally, we analyzed the viability and fitness of the CNS-isolated CD4 T-cells from the spinal cord and brain tissues by examining their ability to proliferate and secrete scFv ex vivo upon restimulation and culture for up to a week. We observed rapid proliferation and up to threefold increase in their cell number after anti-CD3 antibody stimulation over a week of culture (Fig. 6F). Flow cytometry analysis showed that the cell culture supernatant of the scFv-IL-2/CAR-FoxP3 eng.CD4 T-cells bound to CD33 on THP1 cells, confirming scFv secretion ex vivo (Fig. 6G). However, the cell culture supernatant from the control CAR-FoxP3 eng.CD4 T-cells purified from the spinal cord and brain tissues did not bind to THP1 cells (Fig. 6G). These results confirmed that scFv-IL-2/CAR-FoxP3 eng.CD4 T-cells migrate into and persist in the CNS and deliver scFv locally. The data also demonstrated evidence for the survival and fitness of scFv-IL-2/CAR-FoxP3 eng. CD4 T-cells in vivo in the CNS for many weeks.

## Discussion

The blood-brain barrier poses an almost insurmountable obstacle to the treatment of neurodegenerative disorders with large-molecule therapeutic agents such as antibodies. The successful delivery of antibodies into the brain after systemic administration has been limited due to the barriers to these large, highly charged molecules crossing the blood-brain barrier (Zhang et al, 2023). Systemically administered antibody therapeutics designed to neutralize or clear Aβ42 peptide in the CNS have gained only moderate success as potential therapeutic approaches to AD (Shi et al, 2022). However, CAR-T cells could be long-lived in the CNS for months to years for use in a disease that progresses over many years (Wakim et al, 2010; Prasad and Lokensgard, 2019). While CAR T-cell therapy has primarily been used in cancer treatment outside of the CNS, our research explores its potential in the realm of neurodegenerative diseases, including Alzheimer’s Disease, offering a fresh perspective on AD therapeutics or other slowly progressing diseases of the CNS.

CNS CD33 also has emerged as a potential target for therapeutic mediation in AD (Griciuc et al, 2013, 2020), because downregulation of CD33 is associated with a reduction in the amyloid plaque burden in the brain. CD33 is an inhibitory siglec protein, and its blockade by antibody or internalization into cells results in enhanced phagocytic function of microglia and macrophages, including removal of Aβ42 peptide (Griciuc et al, 2013, 2020).

We recently demonstrated that blocking the CD33 using Lintuzumab (HuM195) or its single-chain variable fragment (scFv) increased phagocytosis of Aβ42 peptide in human embryonic stem (hES) cell-derived microglia and human macrophages (Wong et al, 2024). This activation of phagocytosis was driven by antibody binding to CD33, which led to its internalization and degradation (Wong et al, 2024). In addition, blocking CD33 also promoted a secondary immune response via the secretion of IL-33 (Wong et al, 2024) that is associated with reprogramming of a microglia subpopulation to increase Aβ uptake (Xiong et al, 2022). Thus, we provided a mechanistic understanding of the role of CD33 signaling in microglia and macrophages and defined a molecular basis for developing CD33-based therapy for AD (Wong et al, 2024).

Hence, in this study, we introduced a new approach to AD treatment that involves the use of engineered CAR T-cells to deliver anti-CD33 scFv directly and locally into the brain with the goal of enhancing the phagocytosis of Aβ by microglia and brain-borne macrophages. However, unlike in cancer, in neurodegenerative diseases like AD, brain tissue is already undergoing inflammatory cytotoxic processes, so a cell-based drug delivery strategy should not further exacerbate the existing inflammatory pathology (Goel et al, 2022; Lecca et al, 2022; Judd et al, 2023). Additionally, CAR T cells have frequently been used to deliver therapeutic cargo such as enzymes or proteins locally (Peraro et al, 2021; Gardner et al, 2022b). We hypothesized that a non-inflammatory CAR-engineered T-cell, which could home specifically to the brain and secrete an immunoglobulin, might be a proof of concept for such an approach for a brain-targetable cellular micropharmacy (Gardner et al, 2020).

To test this hypothesis, we chose to engineer CD4 T-cells into regulatory-like cells that secrete an anti-CD33 scFv, endow them with CNS homing capacity by using an anti-GD2 CAR, provided them with autologous IL-2 stimulation for growth, and attenuated their inflammatory properties by inserting FoxP3 protein. Successful use of CAR-engineered regulatory like CD4 T-cells would provide dual benefits: 1. FoxP3 could control the ongoing CNS inflammation, and 2. In principle, the use of a GD2-directed CAR that secretes anti-CD33 scFv, the engineered Treg-like cells in the brain could directly activate glia to neutralize the pathogenic molecules such as Aβ or modulate effector function of the neuronal or immune cells without systemic toxicity and without absorption of the CD33 antibody onto the myelomonocytic cells found widely in the blood, bone marrow, liver, and spleen.

Previously, we showed that an scFv directed to CD33 could enhance the phagocytosis of Aβ42 peptide in vitro (Wong et al, 2024). In this work, we demonstrate that similar effects can be achieved by the use of attenuated CD4 CAR T-cells that secrete the scFv in vivo. The cells we created are designed as a proof-of-concept for the use of a cell vehicle to deliver therapeutic agents into the brain. Further optimization along several lines of work would be needed before such a construct would be available for human testing. We detail the construction and expression of the vectors needed to create the cells, the secretion and functional activity of the anti-CD33 scFv cargo (binding to and activation of monocytes to phagocytose Aβ42 peptide) and IL-2 cargo (proliferation of the CAR T cells in vivo), and the functional attenuation of the T cells by FoxP3 transduction. We further show that the cells can be explicitly directed and safely into the brain by use of the GD2 CAR, where they function to secrete scFv and IL-2.

Our research thus takes a unique approach, harnessing the potential of CD4 T-cells, known for their plasticity, to transform into regulatory T-cells under specific conditions. We have developed a one-of-a-kind tool by genetically engineering CD4 T-cells into regulatory-like T-cells. These cells express a cell surface CAR against GD2 antigen, a stable intracellular FoxP3, and secrete IL-2 and a therapeutic cargo protein anti-CD33 scFv. Furthermore, these CAR-engineered Treg-like CD4 T-cells maintain a like phenotype, show suppressive efficacy, and are retained in the brain. Previous studies with CAR or TCR-engineered regulatory T-cells have shown positive outcomes in GvHD, autoimmunity, and chronic inflammation (Fransson et al, 2012; Skuljec et al, 2017; Malviya et al, 2020; Bolivar-Wagers et al, 2022; Qiao et al, 2023). Our approach opens a novel avenue to other diseases in which prolonged delivery of agents into the CNS is needed.

### Outlook

Important challenges to our described approach remain before it might be ready for clinical testing. Most important is the lack of an appropriate model in mice in which to test this concept. Although mouse models of AD and mice with ectopic expression of human CD33 exist (Griciuc et al, 2013, 2020; Eskandari-Sedighi et al, 2024; Su et al, 2025), including human CD33 knock-in model developed by Biocytogen (Waltham, MA), the available mouse models do not provide a suitable solution as the host mice need to be fully immunocompromised to allow engraftment of the engineered human cells and the humanized antibody to CD33 over long periods. Because the disease progresses over 4 to 8 months in the mice, we would have to maintain the cells in the mouse over this period or do repeated injections, which is not possible in immunocompetent mice where rejection of the cells would occur within days; in addition, the CD33 antibody would be quickly neutralized. Hence, at this stage, this work is a proof of concept with more work to be done. Other hurdles include efficiently transferring multiple genes into CD4 T-cells; here, we have demonstrated a proof of concept by successfully transferring five genes using two retrovirus transduction systems, though the process may be problematic and inefficient for large-scale production. Various methods have been developed to enable robust expression of multiple genes while maintaining a reasonable vector size (Kriz et al, 2010; Kebriaei et al, 2017; Patel et al, 2021). In addition, over longer periods of time, T-cells are likely not to be optimal as the preferred delivery vehicle due to their intrinsic inflammatory properties. It is crucial that other cell types (for example, B cells or monocytes) or other mechanisms for durably regulating T cell activities be explored (McCarthy et al, 2012). Finally, while we chose GD2 as our CAR target for this proof, other CNS-specific targets may be more appropriate (McCarthy et al, 2012). The studies described here provide a foundation upon which more optimal cellular delivery vehicles might be constructed for a variety of neurodegenerative disorders requiring long-term treatment inside the CNS.

## Methods

**Table:** Reagents and tools table

Reagent/resource	Reference or source	Identifier or catalog number
**Experimental models**		
NSG mice (NOD.Cg-Prkdcscid Il2rgtm1Wjl/SzJ)	Jackson Lab	**IMSR_JAX:005557**
**Recombinant DNA**		
human FoxP3	Addgene	13250
human IL-2	Addgene	90513
human TGFβ	Addgene	98377
GLuc-mCherry	Addgene	178185
T2A and P2A	Addgene	136477
pSF-CMV-Puro-IL-2	Sigma-Aldrich	OGS1497
GD2-E101K CAR	Gift of Dr. Michael Milone lab	
Anti-CD33 scFv	This study	“Methods”
**Antibodies**		
Amphiregulin-PeCy7	Invitrogen	25-5370-42
CD3-BV510	BioLegend	317331
CD3- BV785	BioLegend	317329
CD3- BV711	BD	563725
CD4 (BV421)	BD	562425
CD4 (PE)	BD	561843
CD4 (AF700)	*BD*	561030
CD8 (AF700)	BD	561026
CD8 (BV786)	BD	563824
CD11b (AF488)	*BD*	557672
CD11b (PerCP-Cy5.5)	BioLegend	301417
CD14 (APC-C7)	BioLegend	301819
CD14 (BV786)	BD	563699
CD14 (PE)	BD	562691
CD25 (PerCPCy5.5)	BioLegend	302626
CD25 (APC)	BioLegend	302610
CD33 (FITC)	BioLegend	303304
CD33(PeCy7)	BD	333949
CD44 (FITC)	BD	347943
CD44 (BV421)	BioLegend	338809
CD44 (APC)	BioLegend	397505
CD45 (FITC)	BioLegend	982316
CD45 (APC-C7)	BD	557833
CD45 (BV650)	BD	560973
CD69 (APC)	BioLegend	310909
CD69 (FITC)	BioLegend	310904
CD69 (BV421)	BioLegend	310929
CD127 (BV785)	BioLegend	351329
CTLA-4 (BV785)	BioLegend	369623
CTLA-4 (APC)	BioLegend	369611
CTLA-4 (PeCy5)	BD	561717
FoxP3(AF647)	BD	560045
FoxP3(V450)	BD	560459
FoxP3(AF488)	BioLegend	320112
GARP (BV421)	BioLegend	352509
GARP (PE)	BioLegend	352503
GARP (PerCP-Cy5.5)	BioLegend	352513
GITR (PerCP-Cy5.5)	BioLegend	371217
GM-CSF (AF647)	BD	562257
Granzyme B (AF647)	BD	561999
Helios (AF488)	BD	563950
Helios(PE)	BD	563801
His6-tag (AF647)	Invitrogen	MA1-21315-A647
ICOS (BV510)	BioLegend	313525
ICOS (PerCP-Cy5.5)	BioLegend	313517
IL-2 (FITC)	BioLegend	500305
IL-4 (APC)	BioLegend	500811
IL-6 (PE)	BioLegend	501106
IL-10 (APC)	BD	554707
IL-12 (AF647)	BioLegend	501818
IL-13 (PE)	BioLegend	501903
IL-17A (AF700)	BD	560820
IFNγ (APC)	BD	562017
LAG-3 (FITC)	Invitrogen	11-2239-41
Myc-tag (AF647)	Cell Signaling	2233S
Myc-tag (AF488)	Cell Signaling	2279S
Myc-tag (PE)	Cell Signaling	3739S
Neuropilin-1 (BV421)	BD	565954
OX40 (FITC)	BioLegend	350006
PD-1 (PeCy7)	BD	561272
PD-1 (BV510)	BioLegend	367423
TGFβ (PerCP-Cy5.5)	BD	562423
TGFβ (BV421)	BD	562962
TGFβ (AF488)	BD	562545
TIGIT (AF700)	Invitrogen	56-9500-41
TIGIT (BV510)	BioLegend	372737
TIM-3 (FITC)	Invitrogen	11-3109-41
TNFα (AF700)	BD	561023
Viability Dye (eFluor 780)	Invitrogen	65-0865-14
Viability Dye (DAPI)	Thermo Scientific	62248
2 A peptide (AF647)	Novus Biologicals	NBP2-59627AF647
APC–anti-GD2 CAR (clone 1A7)	Creative Biolabs	NA
HuM195 antibody	Actinium Pharmaceuticals	NA
anti-RSV antibody	Tri-Institutional Therapeutics Discovery Institute	NA
anti-HPV	CreativeBiolabs	MOB-510-S(P)
Anti-CD3 antibody	Miltenyi Biotec	130-093-387
anti-2A peptide antibody	Novus Bio	NBP2-59627
Goat anti-mouse HRP antibody	R&D Systems	HAF007
**Oligonucleotides and other sequence-based reagents**		
human Aβ42 peptide	Thermo Scientific	AAJ66387MCR
T2A peptide	Genscript	
**Chemicals, enzymes, and other reagents**		
Human IL-2	Peprotech	200-02
Polybrene	Sigma-Aldrich	TR-1003
Retronectin	TaKaRa Biomedicals	T100A
TGFβ	Peprotech	100-35-01 M
all-trans retinoic acid	Sigma-Aldrich	R2625
Lenti-X concentrator	Takara Bio	631232
Human IL-2 ELISA Kit	Abcam	ab270883
ECL	Thermo Scientific	34095
Isoflurane	Covetrus	11695067772
pHrodo™ Green STP Ester	Thermo Scientific	P35369
pHrodo™ Red, succinimidyl ester	Thermo Scientific	P36600
Human Aβ42 peptide	Thermo Scientific	AAJ66387MCR
Latrunculin-A	Cayman chem	10010630
TRIzol	Ambion	15596026
CellTrace Violet (CTV) dye	ThermoFisher	C34571
coelenterazine	NanoLight	3031
**Software**		
Graphpad Prism 10		
FlowJo		
R programming software		
**Other**		
Amersham Imager 600		
Cytation 5 (Biotek)		
Bioluminescence imaging		
BD LSRFortessa Cell Analyzer		
Confocal microscopy		

### Mice

NSG mice (NOD.Cg-Prkdcscid Il2rgtm1Wjl/SzJ), 8–12 weeks old, male and female, were obtained from Jackson Laboratory (Strain #005557). All animal experiments were performed in compliance with MSKCC IACUC-approved protocol 96-11-044.

### Antibodies for flow cytometry

Amphiregulin (PeCy7 - Invitrogen# 25-5370-42), CD3 (BV510, BV785 - BioLegend #317331, #317329; BV711- BD #563725), CD4 (BV421, PE, AF700 - BD #562425, #561843, #561030), CD8 (AF700, BV786 - BD #561026, #563824), CD11b (AF488 - BD #557672; PerCP-cy5.5 - BioLegend #301417), CD14 (APC-C7 - BioLegend #301819; BV786, PE - BD #563699, # 562691), CD25 (PerCp-Cy5.5, APC - BioLegend #302626, #302610), CD33 (FITC - BioLegend #303304; PeCy7 - BD# 333949), CD44 (FITC - BD# 347943; BV421, APC - BioLegend #338809, #397505), CD45 (FITC - BioLegend #982316; APC-C7, BV650, - BD # 557833, #560973), CD69 (APC, FITC, BV421 - BioLegend #310909, #310904, #310929), CD127 (BV785 - BioLegend #351329), CTLA-4 (BV785, APC - BioLegend #369623, #369611; PeCY5 - BD# 561717), FoxP3 (AF-647, V450 - BD #560045, #560459; AF488 - BioLegend #320112), GARP (BV421, PE, PerCP-Cy5.5 - BioLegend #352509, #352503, # 352513), GITR (PerCP-Cy5.5 - BioLegend #371217), GM-CSF (AF647 - BD #562257), Granzyme B (AF647 - BD #561999), Helios (AF488, PE - BD#563950, BD#563801), His6-tag (AF647 - Invitrogen#MA1-21315-A647), ICOS (BV510, PerCP-Cy5.5 - BioLegend #313525, #313517), IL-2 (FITC - BioLegend #500305), IL-4 (APC - BioLegend #500811), IL-6 (PE - BioLegend #501106), IL-10 (APC - BD #554707), IL-12 (AF647 - BioLegend #501818), IL-13 (PE - BioLegend #501903), IL-17A (AF700 - BD #560820), IFN*γ* (APC - BD #562017), LAG-3 (FITC - Invitrogen# 11-2239-41), Myc-tag (AF647, AF488, PE - Cell Signaling #2233S, #2279S, #3739S), Neurophilin-1 (BV421 - BD #565954), OX40 (FITC - BioLegend #350006), PD1 (PeCy7 - BD# 561272; BV510 - BioLegend #367423), TGFβ (PerCp-Cy5.5, BV421, AF488 - BD# 562423, #562962, #562545), TIGIT (AF700 - Invitrogen#56-9500-41; BV510 - BioLegend #372737), TIM-3 (FITC - Invitrogen# 11-3109-41), TNF*α* (AF700 - BD #561023), Viability (Dye eFluor780 - Invitrogen#65-0865-14; DAPI - Thermo Scientific# 62248), 2A peptide (AF647 - Novus#NBP2-59627AF647), APC-anti-GD2 CAR (clone 1A7, creative Biolabs). All primary antibodies were used at a dilution of 1:100. All secondary antibodies were used at a dilution of 1:5000.

### Peptides

The human Aβ42 peptide was purchased from Thermo Scientific (#AAJ66387MCR). The T2A peptide (EGRGSLLTCGDVEENPGP) was synthesized at Genscript (Purity: 96.6%).

### Anti-CD33 scFv, HuM195, control IgG, and control scFv

The clinical grade HuM195 antibody was made available from Actinium Pharmaceuticals, Inc. Anti-CD33 scFv was constructed from the HuM195 antibody sequences (Co et al, 1992). The control IgG (anti-RSV) was generously donated by The Tri-Institutional Therapeutics Discovery Institute, New York. The control scFv (anti-HPV) was purchased from CreativeBiolabs (#MOB-510-S(P)).

### Expression vectors

All of the constructs (scFv-T2A-mCherry, scFv-T2A-IL-2-P2A-mCherry, CAR, CAR-T2A-FoxP3, CAR-T2A-FoxP3-P2A-TGFβ, GLuc-mCherry, and mCherry) were synthesized after codon optimization for human T-cell expression at Genscript and cloned into the SFG-*γ* retroviral vector between 5’LTR and 3’LTR (Jaspers et al, 2023). The anti-CD33 scFv construct was generated by joining the paired heavy and light chain variable region sequences of HuM195 antibody in the order of VH-(G4S)3-VL-T2A-mCherry. The affinity-enhanced anti-GD2-E101K CAR gene sequences were obtained from Dr. Michael Milone’s lab at the University of Pennsylvania (Richman et al, 2018). The human FoxP3 gene (#13250), human IL-2 (#90513), human TGFβ (#98377), GLuc-mCherry (#178185), T2A and P2A (#136477) gene sequences were obtained from the Addgene online database. In addition, anti-CD33 scFv was cloned into a pSF-CMV-Puro-IL-2 secretion plasmid (Sigma-Aldrich) with a C-terminal 8xHis-tag. The 8xHis-tagged scFv was purified from the supernatant of FreeStyle 293 cells using a His column on FPLC as described previously (Wong et al, 2024).

### Isolation of T-cells, Tregs, and monocytes from human PBMC

Human PBMC was prepared by density gradient centrifugation using Corning-Lymphocyte Separation Medium (# 25-072-CV) of fresh blood collected from donors or leukopaks purchased from the New York Blood Center. The human Treg isolation kit (Miltenyi Biotec #130-091-301) was used to isolate CD4 + T-cells and nTregs (CD4 + CD25 + ) sequentially. CD14+ monocytes were isolated from PBMC using the Miltenyi kit (#130-050-201). All experiments with human donor blood were conducted under MSK IRB-approved protocols after written informed consent.

### Production of retrovirus, lentivirus, and CD4 T-cell transduction

The H29/Galv9 systems (Biovec) were used to make stable retrovirus-producing cell lines. CD4 T-cells isolated from fresh human blood or leukopak were stimulated with 3 μg/ml anti-CD3 antibody (Miltenyi Biotec # 130-093-387) and 100 IU of human IL-2 (Peprotec). The activated CD4 T-cells were cultured in a complete medium (RPMI 1640 medium with 10% FBS, 100units/mL penicillin, 100 mg/mL streptomycin) and transduced on days 2 and 3 after isolation. The transduction process involved the mixing of CD4 T-cells with 0.45μm sterile syringe-filtered Galv9 retrovirus supernatant premixed with 5 μg/ml polybrene infection reagent (Sigma) and followed by centrifugation at 2000×*g* for 2 h at 32 °C in six-well plates pre-coated with 10 μg/ml RetroNektin (TaKaRa Biomedicals). For the double retrovirus transduction of CD4 T-cells, the first transduction was performed with CAR-FoxP3 or CAR retroviral supernatant fluid on day 2 post-CD4 T-cell stimulation, followed by a second retrovirus transduction with constructs such as scFv-IL-2-mCherry or GLuc-mCherry the next day. Cell surface expression of CAR and intracellular expression of mCherry confirmed the transduction of CD4 T-cells. Transduced CAR-FoxP3 CD4 T-cells were cultured in RPMI complete media supplemented with 10 ng/ml TGFβ (Peprotech), 10 nM all-trans retinoic acid (Sigma-Aldrich), and 500 IU IL-2 (Peprotech) for 3 days before being used for the in vitro and in vivo experiments.

In experiments where lentivirus transductions were also utilized, viruses were generated using Lenti-X 293T (Lenti X) cells. Briefly, Lenti-X cells were plated in poly-D-lysine-coated 10-cm tissue culture-treated plates. 24 h later, the cells were transfected with PAX2, MD2.G, and lentivirus plasmids at equimolar concentrations using Lipofectamine and P3000 in Opti-MEM media. The media was replaced 24 h later, and viral supernatants were collected at 48 and 72 h post-transfection. The pooled supernatants were filtered with a 0.45-μm filter and concentrated using Lenti-X concentrator (Takara Bio, #631232). The viral pellets were then stored at −80 °C.

### ELISA from T-cell culture supernatant fluid and mouse CSF

Live CD4 T-cells transduced with retrovirus were sorted by flow cytometry alongside untransduced cells. Isolated cells were restimulated with 1 μg/ml anti-CD3 antibody (Miltenyi Biotec #130-093-387) and cultured in a 5–10 ml volume of serum-free Opti-MEM™ medium at 1 million cells/ml concentration (Gibco #31985062). Seventy-two hours later, supernatant fluids were collected for ELISA and western blot. IL-2 ELISA was done using a Human IL-2 ELISA Kit (ab270883). Anti-2A peptide direct-ELISA was done to detect the cell-secreted peptides, scFv, and IL-2. We utilized the 18-amino-acid-long T2A peptide (EGRGSLLTCGDVEENPGP) synthesized at Genescript (Purity: 96.6%) to prepare the known concentration of the standard curve. In brief, a polystyrene flat-bottom 96-well plate (IMMULON 4HBX # 3355) was coated overnight with the known concentrations of T2A peptide or cell supernatant. The next day, an anti-2A peptide-specific primary antibody was incubated for 2 h, followed by an HRP-linked anti-mouse secondary antibody for 1 h at room temperature. Wells were washed five times at each step, and signals were detected using a TMB substrate solution. ELISA from the mouse CSF samples was also performed using the above protocol.

### Western blot from T-cell culture supernatant fluid

After 72 h, T-cell culture supernatant was collected, as mentioned in the ELISA section. Supernatants were concentrated to 1–2 ml using an Amicon Ultra Centrifugal Filter, ten kDa MWCO (# UFC801008). Concentrated supernatant proteins were reduced using Bio-Rad Laemmli Sample Buffer supplemented with 50 mM of DTT and denatured at 90 °C for 5 min. Around 5 μg of concentrated supernatant proteins were loaded on 4–15% SDS-PAGE, transferred to the Nitrocellulose Membrane, and blotted with mouse anti-2A peptide antibody (Novus Bio # NBP2-59627). A secondary HRP-conjugated Goat anti-mouse HRP antibody (R&D Systems, #HAF007) was used to detect the signal with ECL (Thermo Scientific # 34095). Blots were imaged and analyzed on an Amersham Imager 600 to detect the signal with ECL. Blots were imaged and analyzed on an Amersham Imager 600.

### In vitro binding and competition assays

After 3–5 days, supernatant fluid of scFv-secreting or control CD4 T-cells were incubated on ice with THP1 or human monocytes for 30 min. The supernatant fluid was removed, and cells were washed with FACS buffer (PBS + 2% FBS + 5 mM EDTA+ 1× Pen/Strep) before incubating with an APC-labeled anti-2A peptide antibody for 30 min on ice. APC-positive cells were analyzed by flow cytometry.

For the CD33 competition binding assay, THP1 or human monocytes were pre-incubated with HuM195 or control IgG (1 μg/ml) for 10 min on ice before washing and incubating with the supernatant fluid of scFv-secreting CD4 T-cells. CD33-bound scFv was stained with APC-labeled anti-2A peptide and analyzed by flow cytometry.

### In vivo binding assay in the peritoneal cavity

NSG mice were injected with two million control or scFv-secreting CD4 T-cells into the peritoneal cavity; 48 h later, one million THP1 cells were injected into the peritoneal cavity. Thirty minutes later, mice were euthanized with CO_2_. Injected cells were isolated by peritoneal lavage, and THP1 cell-bound scFv was analyzed by flow cytometry on the CD45 + CD3- population. Absolute cell numbers were derived by volumetric scaling: events were acquired from a defined 100 µL aliquot of the resuspended sample, and the counted pHrodo+ Hu‑Mø events were multiplied by the total suspension volume to estimate the absolute number of pHrodo+ cells recovered from the peritoneal cavity.

### Central nervous system in vivo binding assay

Human PBMCs were isolated from a leukopak as detailed above, followed by CD4 T cell isolation using the Easysep Human CD4 T cell isolation kit (Stemcell Technologies, #17952). Isolated CD4 T cells were stimulated with Immunocult CD3/CD28/CD2 T cell activator (Stemcell Technologies, #10970) and human IL-2 (10 ng/ml) on Day 0. Following this, CD4 T cells were cultured in Immunocult-XF T cell expansion medium (Stemcell Technologies, #10981) with fresh IL-2 supplemented every 2 days as detailed in the protocol. CD4 T cells were transduced with either αCD33-T2A-mcherry or mcherry-only construct on Day 3 with retrovirus; on Day 4, the cells were transduced with αGD2 CAR using lentivirus. Four days later, CD4 T cells were sorted for either αGD2 CAR and CD33-mCherry double-positive cells, or αGD2 CAR and mCherry-only control cells.

On Day 12, 3 million αGD2 CAR CD4 T-cells either secreting αCD33 scfv or mcherry control were injected intracisternally into NSG mice between 6 and 8 weeks of age (*n* = 9 per group). Mice were anesthetized in an insulated chamber perfused with 2–3% isoflurane (Covetrus, 11695067772) in medical air. Mouse hair was removed from the injection site, and the area was sterilized three times with ethanol. For intracisternal injection, 10 μl of cancer cell suspension in PBS was introduced into the cisterna magna using a Hamilton syringe (Hamilton, HT80501) fitted with a 30 G needle, as described previously with minor modification (REF PMID 28283064). In brief, the mouse was positioned prone over a 15-ml conical tube for maximal cervical spine flexion, the occiput was palpated, the needle was advanced 4 mm deep and the syringe content was slowly released into the cisterna magna. The syringe was then held in this position for another ten seconds and then carefully ejected to prevent the reflux of injected liquid. 48 h later, 3 M THP1 cells expressing GFP were again injected intracisternally. An hour later, mice were euthanized with CO_2_, and their brains were harvested. To isolate the cells in the cerebrospinal fluid (CSF), the cranial vault was opened, and the brain was removed. The inner cranial vault and basilar meninges were washed with PBS four times (1 ml each time) in a 6 cm dish. Following this, the surface of the brain and ventricles were washed with 1 ml PBS 4–5 times. All CSF washes were combined in a 15-ml tube along with the corresponding brain to recover any remaining cells. The collected cells were pelleted and lysed with ACK lysis buffer. THP1 cell-bound scFv from respective groups was analyzed via flow cytometry using the α2A-peptide antibody by specifically gating on GFP+ cells.

### Absolute quantification of engineered T cells recovered from CNS compartments

At designated endpoints, mice were euthanized with CO_2_, perfused with PBS to remove circulating cells, and CSF, spinal cord, and brain were collected. Tissues were mechanically dissociated, processed with myelin/debris removal, and resuspended to a known final volume. Flow cytometry acquisitions were performed on a defined 100 µL (0.1 mL) aliquot under identical instrument settings across groups. Human engineered T cells were identified as live singlets (DAPI − ), human CD45 + , CD4 + , mCherry+ (reporter), and CAR+ (surface). Absolute counts per CNS compartment were calculated by volumetric scaling: counted CD45 + CD4+ mCherry+ CAR+ events in 100 µL × (total resuspension volume/0.1 mL). When counting beads were included, bead normalization was applied: engineered T-cell events × (total beads added/beads acquired) × (total sample volume/volume analyzed).

### pH-sensitive Rhodamine (pHrodo)-Aβ42 conjugation

Thermofisher’s pHrodo™ Green STP Ester (#P35369) and the pHrodo™ Red, succinimidyl ester (#P36600) were used to conjugate the Human Aβ42 peptide (Thermo Scientific #AAJ66387MCR) following the supplier’s instructions with slight modifications as described previously (Wong et al, 2024).

### In vitro live imaging of pHrodoRed-Aβ42 phagocytosis

Primary human macrophages or THP1 cell-derived macrophages (20,000) were seeded in 96 wells and pre-incubated with either PBS (no treatment), purified anti-CD33 scFv (1 µg/ml), positive control HuM195 (1 µg/ml), control scFv (1 µg/ml), control IgG (1 µg/ml), or 1 µM Latrunculin-A, a phagocytic inhibitor (Cayman chem # 10010630), as a negative control for 4 h before the addition of 1 µM pHrodoRed-Aβ42. Images were continuously taken every 2 h with brightfield and RFP channel (10x objective) using Cytation 5 (Biotek). Continuous live imaging was quantified with the Gen5 software, and RFP intensity (A.U.) was measured after masking for individual cells.

### Differentiation of primary human monocytes and THP1-cells into macrophages

Primary human CD14+ monocytes were cultured with M-CSF (50 ng/ml), IL-4 (100 ng/ml), and IL-13 (100 ng/ml) cytokines in RPMI complete media. Half of the old culture media was replaced every 2–3 days with fresh media supplemented with the above cytokines. Differentiation of human monocytes into macrophages (Hu-Mø) was monitored under a microscope, confirmed by the changes in their shape and cell surface protein expression, and characterized by flow cytometry analysis. However, THP1 cell-derived macrophages (THP1-Mø) were generated by treating THP1 cells with phorbol 12-myristate 13-acetate (PMA) and ionomycin-cell stimulation cocktail (eBioscience # 00-4970-03). Hu-Mø and THP1-Mø were generated as adherent cells for in vitro studies and as suspension cells when required for in vivo injection.

### In vivo phagocytosis assay

NSG mice were injected with two million control or scFv-secreting CD4 T-cells and one million human primary macrophages or THP1-Mø and pHrodoGreen-Aβ42 (200 μg) into the intraperitoneal cavity. Seventy-two hours later, mice were euthanized with CO_2_. Injected cells were isolated by peritoneal lavage, and phagocytosis of pHrodoGreen-Aβ42 was analyzed by flow cytometry on the CD45 + CD3- population.

### Differentiation of human stem cells into microglia

Microglial progenitors were derived from the H9 stem cell lines using the protocol published by (Fattorelli et al, 2021). In brief, healthy stem cells at 70–80% confluence were dissociated by Accutase. The cells were spun down and resuspended in BVS (mTeSR1 + 100 µg/ml BMP4, 100 µg/ml VEGF, 100 µg/ml SCF) medium with 10 µM Y-27632 at a final concentration of 100k cells/ml. Using a multi-channel pipette, 110 µl of this cell suspension was added to each well of an ultra-low attachment U-bottom plate. The plate was spun at 100 rcf for 3 min to help cells cluster at the bottom of the plate. This is marked as day 0 of the differentiation process. 75% of BVS media was replaced on D1, D2, and D3, and embryoid body (EB) formation was monitored. On D4, EBs were collected in a Falcon tube, and 8 EBs were plated per well on a six-well plate in 2 ml SMIFT (StemPro-34, 55 µM B-mercaptoethanol, 100 µg/ml SCF, 50 µg/ml mCSF, 100 µg/ml IL3, 100 µg/ml Flt3, 50 µg/ml TPO) medium. SMIFT media was replaced on D8. Media was switched to FMG (100 µg/ml Flt3, 50 µg/ml mCSF, 100 µg/ml GM-CSF) medium on D11. Microglial progenitors accumulate in the media over 1 week. On D18, progenitors were collected by filtering media through a cell strainer to remove EBs. In accordance with the protocol published by Guttikonda et al (Guttikonda et al, 2021), microglia were matured on six-well plates for 1 week in RPMI complete media supplemented with 10% FBS, 50 µg/ml mCSF, and 100 µg/ml IL34 with media changes every alternate day. All recombinants were purchased from R&D.

### Transcriptome sequencing (RNAseq)

CD4 T-cells isolated from human PBMC were transduced to express CAR-FoxP3 or CAR alone. One week post-transduction, CAR-positive CD4 T-cells were sorted by flow cytometry. Isolated cells were restimulated with 1 μg/ml anti-CD3 antibody (Miltenyi Biotec # 130-093-387) and cultured in complete RPMI media supplemented with 10 ng/ml TGFβ (Peprotech), 10 nM all-trans retinoic acid (Sigma-Aldrich), and 500 IU IL-2 (Peprotech) (Schmidt et al, 2016). After 3 days of culture, 1 million cells were frozen in TRIzol reagent (Ambion # 15596026) and submitted to MSK’s Integrated Genomics Operation (IGO) facility for RNA library preparation and sequencing with a depth of 100–120 million reads per sample. MSK’s Bioinformatics Core (BIC) facility analyzed the data for differential gene expression.

### Regulatory T-cells suppression assay

The in vitro Treg suppression assay was done using the previously published protocol (Collison and Vignali, 2011) with slight modifications. Briefly, effector CD3 T-cells (Teff) were isolated from PBMC and labeled with CellTrace Violet (CTV) dye (ThermoFisher # C34571). Fifty thousand CTV-labeled CD3 Teff cells were added per well in a round-bottom 96-well tissue culture plate and cultured alone, with natural Tregs, or Treg-like CAR-FoxP3 engineered CD4 T-cells at different Tregs: Teff ratios and activated with 3 μg/ml of anti-CD3 antibody (Miltenyi Biotec # 130-093-387). After 72 h, the suppressive efficiency of Tregs was determined by flow cytometry analysis of CTV dilution in the responder CD3 Teff cells.

### Flow cytometry staining of cell surface and intracellular proteins

Six days post-transduction, CD4 T-cells were re-stimulated with 1 µg/ml anti-CD3 mAb (Miltenyi Biotec # 130-093-387) and cultured in RPMI complete media supplemented with 10 ng/ml TGFβ (Peprotech), 10 nM all-trans retinoic acid (Sigma-Aldrich), and 500 IU IL-2 (Peprotech). Seventy-two hours post-restimulation, cells were stained for the selected cell surface markers such as CD4, CD8, CD25, CD127, NRP-1, AREG, GITR, PD-1, CTLA-4, ICOS, etc., as well as a viability dye (DAPI or eFluor 780). For intracellular staining, on day 3 post-restimulation, cells were incubated with the protein transport inhibitor GolgiStop for four hours before fixation/permeabilization and intracellular staining for proteins such as FoxP3, Helios, IL-10, IFN*γ*, etc., and analyzed by flow cytometry. Flow cytometry analysis was done on BD LSRFortessa Cell Analyzer.

### In vivo tracking of CAR-FoxP3 eng.CD4 T-cells

We generated the Gaussia luciferase (GLuc) reporter system to track cell retention in vivo by Bioluminescence imaging (BLI). NSG mice were injected with three million double-transduced CAR-FoxP3/GLuc or CAR/GLuc eng.CD4 T-cells, or just GLuc eng.CD4 T-cells intravenously and mice were imaged weekly by the IVIS-BLI system using the intravenous injection of GLuc substrate, 500 µg of water-soluble coelenterazine (NanoLight # 3031).

### Immunofluorescence staining of the mouse brain

NSG mice were engrafted intravenously with three million CAR-FoxP3, CAR eng.CD4 T-cells, or PBS. Twenty-one days after injection, mice were euthanized with CO_2._ Brains were harvested and fixed in 4% PFA overnight. They were then dehydrated in 70% ethanol for 72 h before embedding in paraffin. Immunofluorescence staining was done on FFPE sections using anti-human CD4 antibody and imaged by confocal microscopy.

### Flow cytometry analysis of CNS tissues

NSG mice were engrafted intravenously with three million double transduced scFv-IL-2/CAR-FoxP3 eng.CD4 T-cells or CAR-FoxP3 eng.CD4 T-cells. The CSF, spinal cord, and brain were collected weekly from at least three mice from each group. Accumulated human CD4 T-cells were isolated from the homogenized brain and spinal cord tissues using a tumor dissociation kit (Miltenyi Biotec 130-096-730). Cells isolated from the CNS tissues and CSF samples were analyzed by flow cytometry and cultured for ex vivo assays.

### GvHD and survival assay

NSG mice were engrafted intravenously with three million engineered CD4 T-cells, and mice were monitored weekly for up to 10 weeks for related toxicity such as weight change, GvHD, and death. Mice developing any signs of hunched posture, skin lesions, diarrhea, or death were considered GvHD positive. The Kaplan–Meier graphs were generated for both GvHD and the probability of survival.

### Statistical analysis

Reported significant values were obtained after multiple comparisons performed using two-way and one-way ANOVA by comparing the mean of each group with the mean of every other group using GraphPad Prism 10. The analysis of the nonparametric paired *t* tests was performed for ELISA. For survival and GvHD data, Kaplan–Meier curves were plotted and compared using a log-rank test. Differential gene expression analysis was performed using the R/Bioconductor software package edgeR in the R statistical programming environment (*P < 0.05; **P < 0.01; ***P < 0.001; ****P < 0.0001) (McCarthy et al, 2012; Robinson et al, 2010). Exact *P* values are presented in Appendix Table S1.

**Figure EV4:**
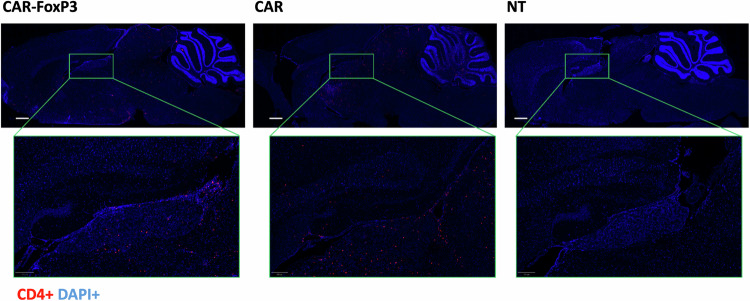
Hippocampus infiltration of CAR-FoxP3 eng.CD4 T-cells. Representative immunofluorescence images of the brain hippocampus regions confirmed the presence of engineered T-cells in both CAR-FoxP3 eng.CD4 T-cells and CAR eng.CD3 T-cells groups but not in the non-transduced (NT) group on day 14 post-injection (*n* = 3 for each group). Representative immunofluorescence staining of CNS-retained human CD4 T-cells in both CAR-FoxP3 eng.CD4 T-cells and CAR eng.CD3 T-cells groups in brain regions proximal to the midrbrain/hypothalamus shown in Fig. 4F. Scale bars, 200 μm.

## Supplementary information

Appendix

Peer Review File

Source data Fig. 2

Source data Fig. 5

Expanded View Figures

## Data Availability

Source data for Figs. 1–6 have been provided. Specifically, source data for Figs. 1, 3, 4, and 6 are available in the following database: BioStudies S-BSST2770. Raw immunofluorescence images of mouse brain sections generated in Fig. 4F and Fig EV4 have also been deposited in BioImage Archive under the accession number S-BSST2770. Raw data files and processed data files for RNA sequencing in Fig. 3C have been deposited in the publicly available Gene Expression Omnibus (GEO) database. The datasets produced in this study are available in the following database: RNA sequencing data: GEO GSE321698. All the other data are also available from the corresponding authors upon request. The source data of this paper are collected in the following database record: biostudies:S-SCDT-10_1038-S44321-026-00421-9.
