# Supplementary material for: Targeted cellular micropharmacies deliver therapeutic agents to the brain
Source: EMBO Mol Med. 2026 Apr 14;18(6):2455–82. doi: 10.1038/s44321-026-00421-9 (PMC13270026; doi:10.1038/s44321-026-00421-9)
Supplement: Supplementary file 1 — Appendix [file 44321_2026_421_MOESM1_ESM.pdf]

APPENDIX

Table of Contents

	Page
Appendix Figure S1	2
Appendix Figure S2	3
Appendix Figure S3	4
Appendix Table S1	5-7

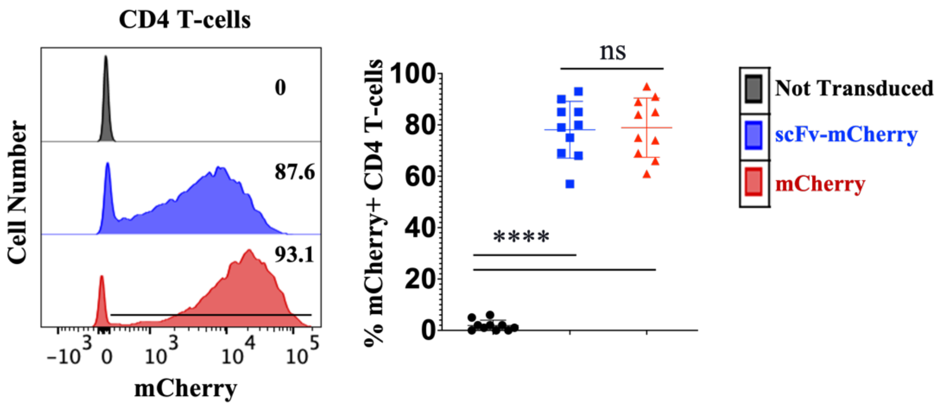

Appendix Figure S1. The transduction efficiency of anti-CD33 scFv retrovirus. Left is a representative histogram showing human primary CD4 T-cells transduced with scFv-mCherry retrovirus (blue), mCherry control retrovirus (red), or untransduced (black). The percentage of mCherry expression represents the transduction efficiency of each virus. In right, the transduction mean with SD of ten experiments are shown. The two-way ANOVA multiple comparisons statistical analysis was performed using Prism 10. Means±SD; n=10. (****p < 0.0001). ns (not significant).

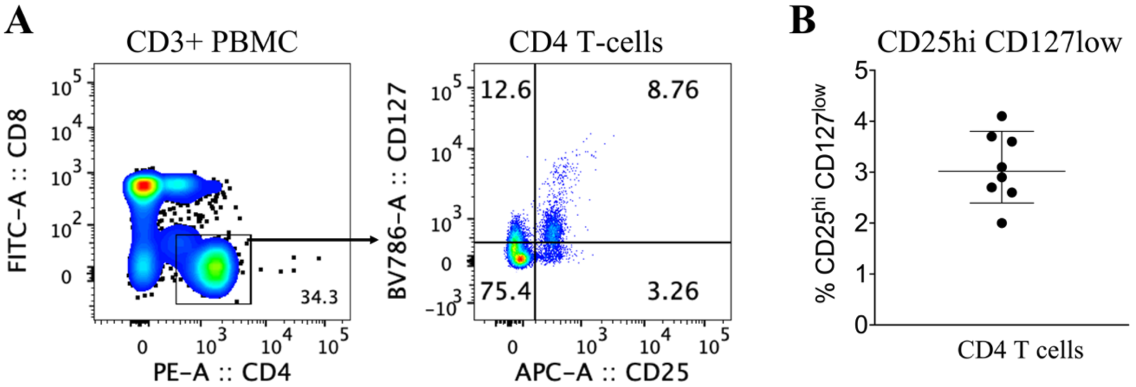

Appendix Figure S2. Percentage of natural Tregs in human PBMCs based on commonly used cell surface markers. (**A**) Showing a representative flow data of CD25hi and CD127low CD4 T-cells in human PBMCs isolated from a leukopak. (**B**) Showing percentage mean and SD of natural Tregs (CD25hi, CD127low) isolated from eight PBMC preparations.

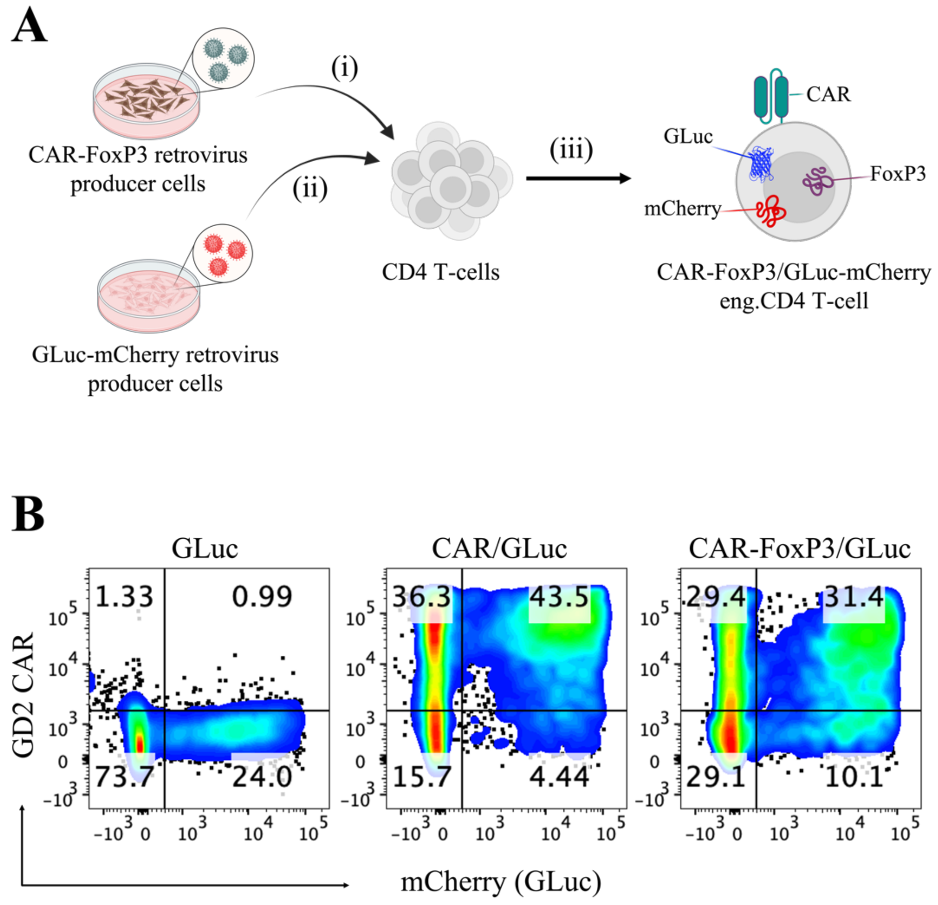

Appendix Figure S3. Dual retrovirus transductions of CD4 T-cells. (A) Schematic diagram showing sequential transductions of human CD4 T-cells with two retroviruses to deliver CAR-FoxP3 and GLuc-mCherry genes, respectively. Both the CAR-FoxP3 retrovirus and the GLuc-mCherry retrovirus were produced separately. Activated CD4 T-cells were first transduced with CAR-FoxP3 retrovirus followed by Gluc-mCherry retrovirus transduction the next day. Double transduction of CD4 T-cells was confirmed by cell-surface CAR staining and intracellular mCherry expression. **(B)** A representative flow cytometry dot plot showing primary human CD4 T-cells double transduced with two retroviruses to express CAR-FoxP3/GLuc-mCherry. Similarly, CD3 T-cells transduced with CAR/GLuc-mCherry, or GLuc-mCherry alone for in vivo toxicity and CNS migration study. To shorten the names, we used the abbreviation GLuc instead of GLuc-mCherry on the top of each dot plot.

Appendix Table S1. Exact p values

Figure	Comparison	Exact p value	Significance
1D	Main comparison	$<1 \times 10^{-4}$	****
2A	Main comparison	2.63×10^{-4}	***
2B	Main comparison	$<1 \times 10^{-4}$	****
3D	Treg:T_{e}ff = 1:1	5.32×10^{-3}	**
3D	Treg:T_{e}ff = 1:2	$<1 \times 10^{-4}$	****
3D	Treg:T_{e}ff = 1:4	$<1 \times 10^{-4}$	****
3D	Treg:T_{e}ff = 1:8	2.09×10^{-3}	**
4E	Main comparison	3.60×10^{-4}	***
4E	GLuc vs. CAR-FoxP3/GLuc	$<1 \times 10^{-4}$	****
4E	GLuc vs. CAR/GLuc	$<1 \times 10^{-4}$	****
5D	NT vs. CAR-FoxP3/scFv-IL-2	0.0298	*
5D	CAR vs. CAR-FoxP3/scFv-IL-2	0.0249	*
5D	NT vs. CAR	3.00×10^{-4}	***
5D	CAR-FoxP3/scFv vs. CAR-FoxP3/scFv-IL-2	8.00×10^{-4}	***
5D	CAR vs. CAR-FoxP3/scFv	$<1 \times 10^{-4}$	****
6B	Main comparisons	0.0078	**
6E	Various comparisons	<0.05	*
6E	Various comparisons	<0.01	**
6E	Various comparisons	<0.001	***
6E	Various comparisons	$<1 \times 10^{-4}$	****
6F	Various comparisons	<0.05	*
6F	Various comparisons	<0.01	**
6F	Various comparisons	<0.001	***
6F	Various comparisons	$<1x10^{-4}$	****
EV1	Main comparison	0.0224	*
EV2C	CAR vs. CAR-FoxP3	0.0072	**
EV2C	CAR vs. CAR-FoxP3-TGFβ	$<1x10^{-4}$	****
EV2C	CAR-FoxP3 vs. CAR-FoxP3-TGFβ	$<1x10^{-4}$	****

EV3	IFNy-Not transduced vs. CAR	0.0242	*
EV3	IFNy-CAR vs. CAR FoxP3	0.0085	**
EV3	IFNy-CAR vs. nTreg	0.0006	***
EV3	IL-17-Not transduced vs. CAR FoxP3	0.0011	**
EV3	IL-17-Not transduced vs. nTreg	$<1x10^{-4}$	****
EV3	IL-17-CAR vs. CAR-FoxP3	0.0012	**
EV3	TNFα-Not transduced vs. CAR-FoxP3	0.0056	**
EV3	TNFα-Not transduced vs. nTreg	0.0006	***
EV3	TNFα-CAR vs.CAR-FoxP3	0.0006	***
EV3	TNFα-CAR vs. nTreg	0.0001	***
EV3	TGFβ-Not transduced vs. nTreg	0.0038	**
EV3	TGFβ-CAR vs. nTreg	0.0122	*
EV3	IL-10-Not transduced vs. nTreg	0.0099	**
EV3	IL-10-CAR vs. nTreg	0.0373	*
EV3	Helios-Not transduced vs. nTreg	$<1x10^{-4}$	****
EV3	Helios-CAR vs. nTreg	$<1x10^{-4}$	****
EV3	Helios-CAR vs. CAR-FoxP3	0.015	*
EV3	Helios-CAR-FoxP3 vs. nTreg	0.0007	***
EV3	FoxP3-Not transduced vs. CAR-FoxP3	0.015	*
EV3	FoxP3-CAR vs. CAR-FoxP3	0.0007	***
EV3	FoxP3-CAR vs. nTreg	$<1x10^{-4}$	****
EV3	FoxP3-Not transduced vs. nTreg	$<1x10^{-4}$	****
EV3	FoxP3-CAR vs. nTreg	0.0002	***
EV3	NRP-1-Not transduced vs. nTreg	0.0012	**
EV3	NRP-1-Not transduced vs. CAR-FoxP3	0.0472	*
EV3	NRP-1-CAR vs. CAR-FoxP3	0.0215	*
EV3	NRP-1-CAR vs. nTreg	0.0006	***
EV3	AREG-Not transduced vs. nTreg	0.0232	*
EV3	AREG-Not transduced vs. CAR FoxP3	0.0496	*
EV3	CD25-Not transduced vs. CAR	$<1x10^{-4}$	****

EV3	CD25-Not transduced vs. CAR FoxP3	<1x10^{-4}	****
EV3	CD25-Not transduced vs. nTreg	<1x10^{-4}	****
EV3	GITR-Not transduced vs. CAR	0.0066	**
EV3	GITR-Not transduced vs. CAR-FoxP3	0.0225	*
EV3	GITR-Not transduced vs. nTreg	0.0163	*
EV3	ICOS-Not transduced vs. CAR	0.0012	**
EV3	ICOS-Not transduced vs. CAR-FoxP3	<1x10^{-4}	****
EV3	ICOS-CAR vs. CAR-FoxP3	0.0033	**
EV3	ICOS-CAR vs. nTreg	0.018	*
EV3	ICOS-CAR FoxP3 vs. nTreg	<1x10^{-4}	****
EV3	CD127-Not transduced vs. CAR	0.0169	*
EV3	CD127-Not transduced vs. CAR-FoxP3	0.0056	**
EV3	CD127-Not transduced vs. nTreg	0.0106	*
EV3	GARP-Not transduced vs. CAR-FoxP3	0.0035	**
EV3	GARP-CAR-FoxP3 vs. nTreg	0.0122	*
EV3	TIGIT-Not transduced vs. CAR-FoxP3	0.0101	*
EV3	TIGIT-Not transduced vs. nTreg	0.0056	**
EV4	scFv, CAR-FoxP3 vs. CAR-FoxP3	0.0005	***
EV4	scFv-IL-2, CAR-FoxP3 vs. CAR-FoxP3	0.0005	***
