# Supplementary figures and images for: Targeted cellular micropharmacies deliver therapeutic agents to the brain
Source: EMBO Mol Med. 2026 Apr 14;18(6):2455–82. doi: 10.1038/s44321-026-00421-9 (PMC13270026; doi:10.1038/s44321-026-00421-9)

## Slide 1
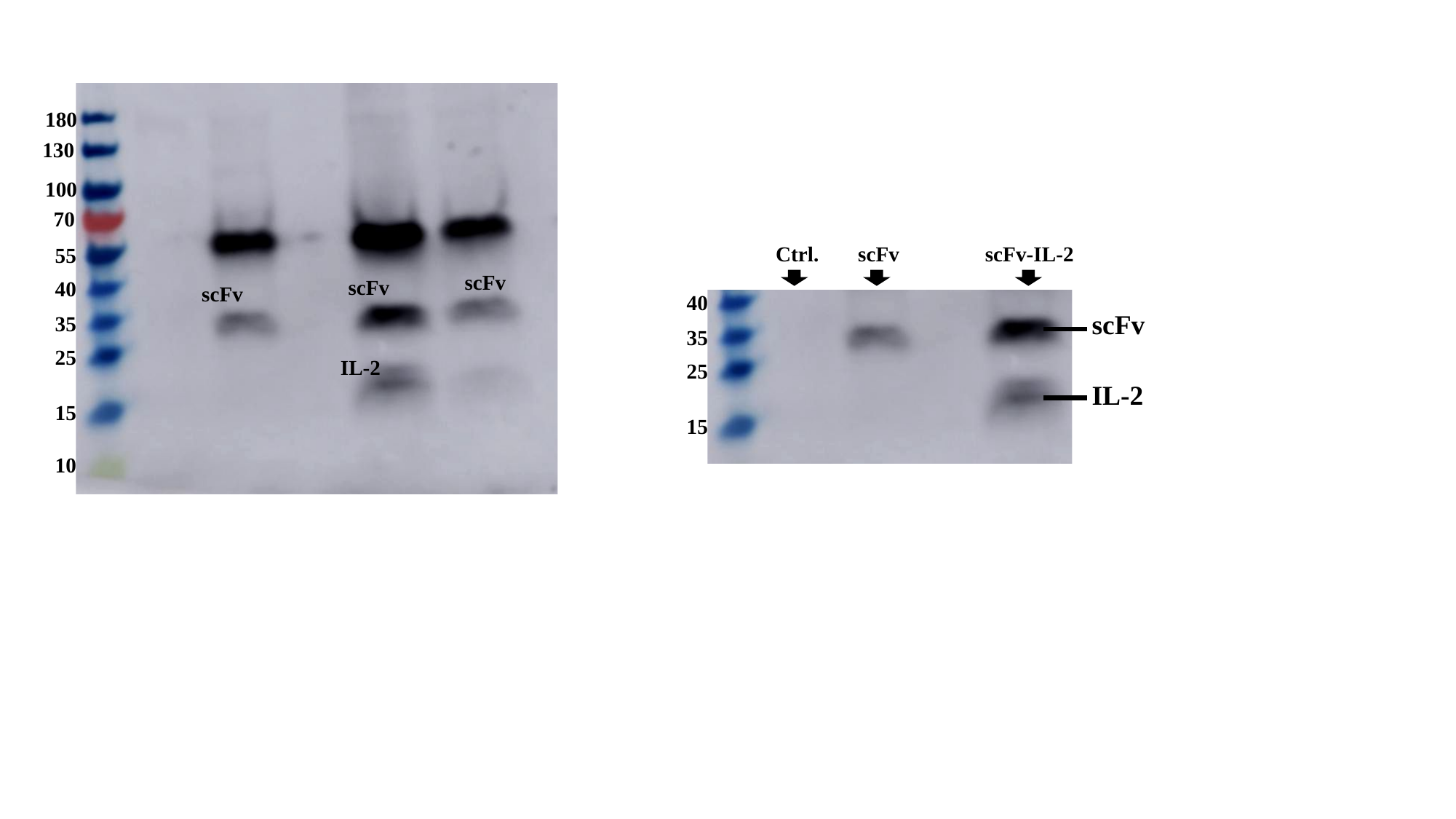

180
130
100
70
Ctrl.
scFv
scFv-IL-2
40
scFv
35
25
IL-2
15
55
scFv
scFv
40
scFv
35
25
IL-2
15
10

Supplement: Supplementary file 4 — Source data Fig. 5 [file 44321_2026_421_MOESM4_ESM.zip › Fig 5/Fig 5C-WB/5C.pptx]
