# Supplementary material for: Targeted cellular micropharmacies deliver therapeutic agents to the brain
Source: EMBO Mol Med. 2026 Apr 14;18(6):2455–82. doi: 10.1038/s44321-026-00421-9 (PMC13270026; doi:10.1038/s44321-026-00421-9)
Supplement: Supplementary file 5 — Expanded View Figures [file 44321_2026_421_MOESM5_ESM.pdf]

Expanded View Figures

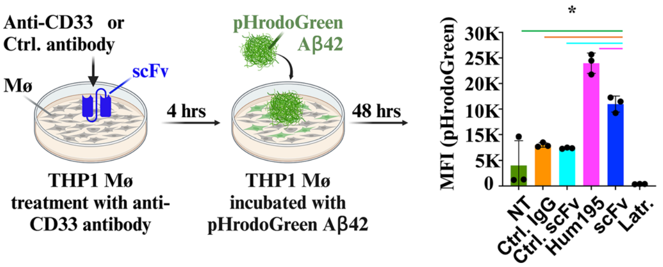

Figure EV1. Anti-CD33 scFv treatment increased Aβ42 uptake by THP1 cell-derived macrophages.

THP1 cell-derived macrophages (THP1-Mø) were generated as described in materials and methods and were pre-treated with: PBS (no treatment; NT), purified anti-CD33 scFv (1 µg/ml), positive control HuM195 (1 µg/ml), control scFv (1 µg/ml), control IgG (1 µg/ml), and Latrunculin as a negative control (1 µM), respectively, for 4 h before pHrodoGreen-labeled Aβ42 (1 µM) was added to the cells to initiate phagocytosis. Forty-eight hours later, cells were detached, and quantification of phagocytosed pHrodoGreen-Aβ42 was analyzed by flow cytometry. The mean fluorescence intensity (MFI) of pHrodoGreen-positive THP1-Mø is presented in the bar diagram. The two-way ANOVA multiple comparisons statistical analysis was performed using Prism 10. means ± SEM; $n = 3$ (*$P < 0.05$).

© The Author(s)

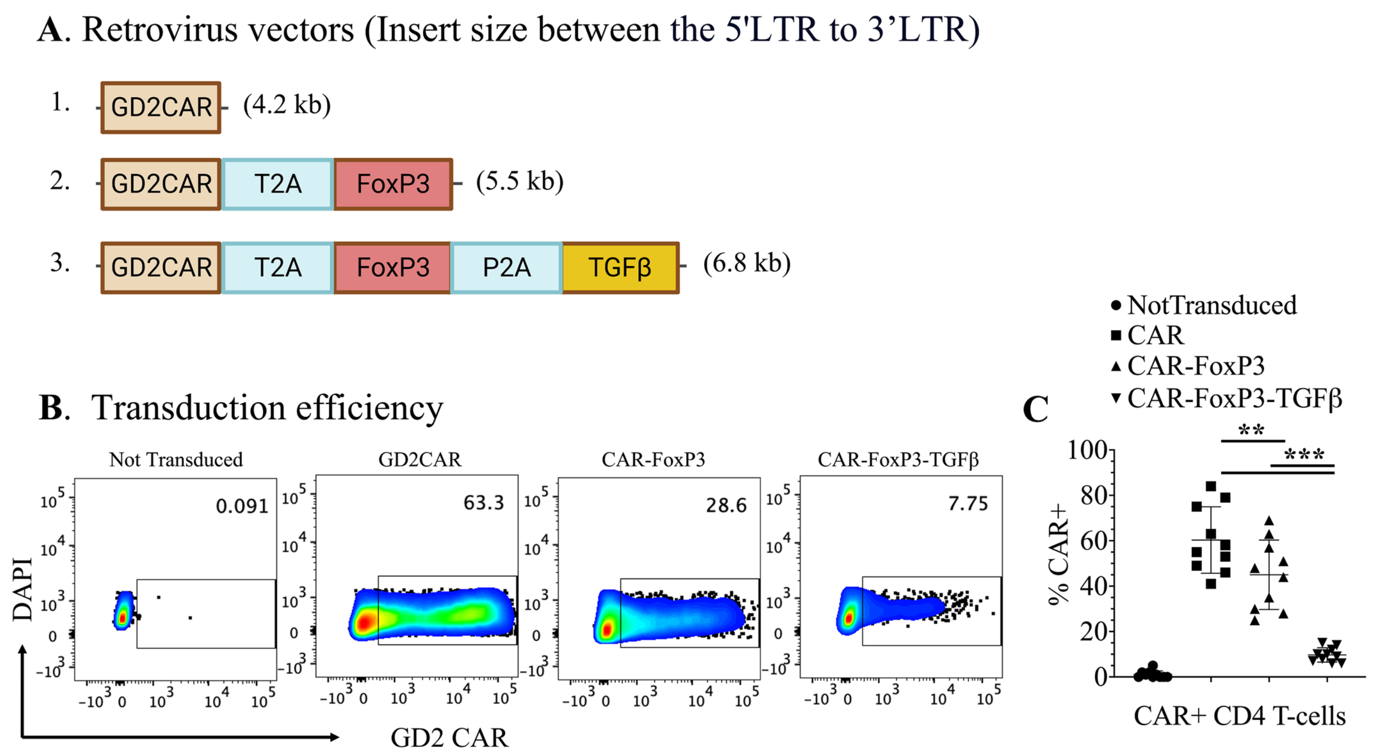

Figure EV2. Transduction efficiency of mono-cistronic, bi-cistronic, and tri-cistronic retroviral constructs.

(**A**) SFG retrovirus vectors were constructed expressing: 1. CAR alone (mono-cistronic), 2. CAR-FoxP3 (bi-cistronic), 3. CAR-FoxP3-TGFβ (tri-cistronic) between their 5′LTR and 3′LTR regions. (**B**) Representative dot plots show transduction efficiency of respective retroviral vectors as percent CAR-positive CD4 T-cells on D7 post-transduction. (**C**) Showing percent transduction mean with SD of ten experiments. The Two-way ANOVA multiple comparisons statistical analysis was performed using Prism 10 (** $P < 0.01$; *** $P < 0.001$).

 © The Author(s)

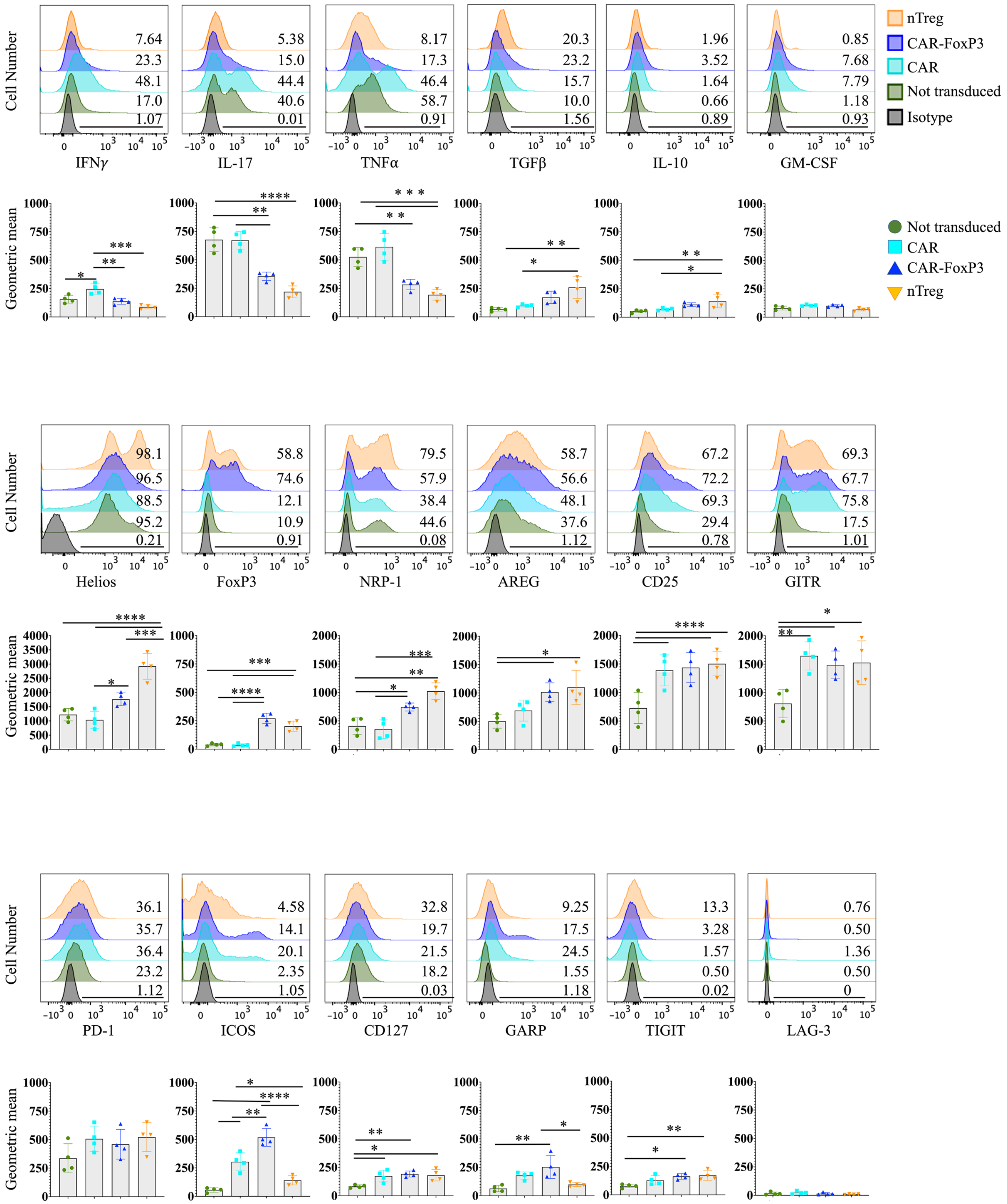
Cell Number
Geometric mean
IFNγ
IL-17
TNFα
TGFβ
IL-10
GM-CSF
nTreg
CAR-FoxP3
CAR
Not transduced
Isotype
Helios
FoxP3
NRP-1
AREG
CD25
GITR
PD-1
ICOS
CD127
GARP
TIGIT
LAG-3

◀ **Figure EV3. Intracellular and cell surface protein characterization.**

Representative overlaid histograms showing the expression of intracellular and cell surface markers among the indicated T-cells. Six days post-transduction, flow cytometry-sorted CAR-positive CD4 T-cells (CAR and CAR-FoxP3), untransduced CD4 T-cells, and nTreg were restimulated with anti-CD3 antibodies and cultured in media supplemented with TGFβ, all-trans retinoic acid, and IL-2. Seventy-two hours post-stimulation, cells were stained for the selected cell surface markers. For intracellular staining, on day 3 post-restimulation, cells were incubated with the protein transport inhibitor GolgiStop for four hours before fixation/permeabilization and intracellular staining and analyzed by flow cytometry. The bar diagram below each histogram shows the geometric mean with SD of the respective staining from four independent stainings. The two-way ANOVA multiple comparisons statistical analysis was performed using Prism 10 (*$P < 0.05$; **$P < 0.01$; ***$P < 0.001$).

 © The Author(s)

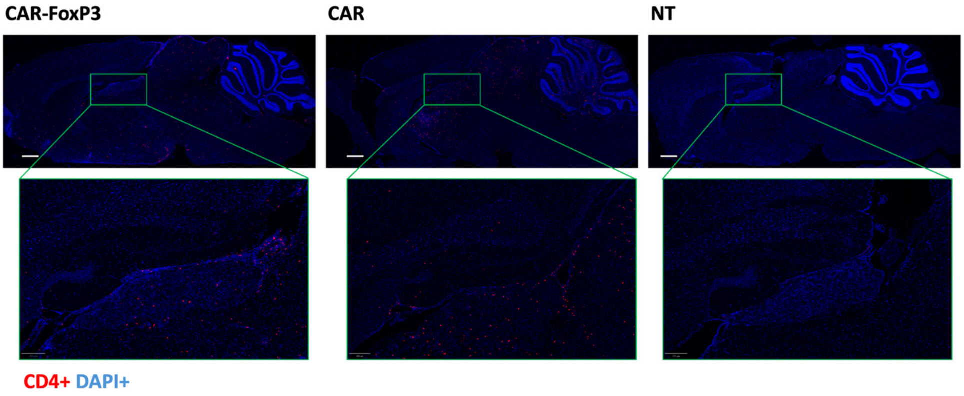

Figure EV4. Hippocampus infiltration of CAR-FoxP3 eng.CD4 T-cells.

Representative immunofluorescence images of the brain hippocampus regions confirmed the presence of engineered T-cells in both CAR-FoxP3 eng.CD4 T-cells and CAR eng.CD3 T-cells groups but not in the non-transduced (NT) group on day 14 post-injection ($n = 3$ for each group). Representative immunofluorescence staining of CNS-retained human CD4 T-cells in both CAR-FoxP3 eng.CD4 T-cells and CAR eng.CD3 T-cells groups in brain regions proximal to the midrbrain/hypothalamus shown in Fig. 4F. Scale bars, 200 µm.

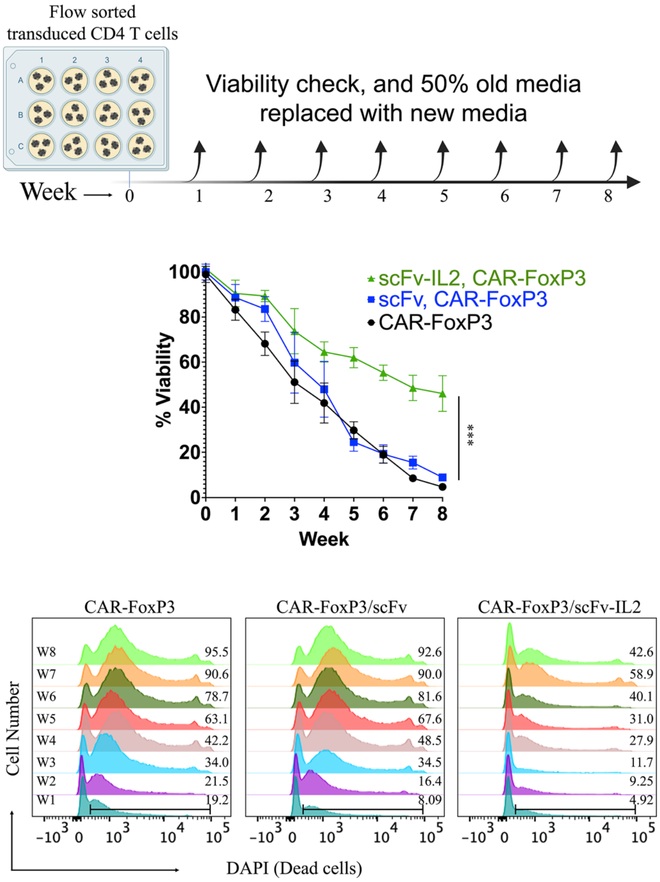

Figure EV5. In vitro long-term survival of eng. CD4 T-cells in IL-2-free culture media.

Human primary CD4 T-cells were double transduced to express scFv-IL-2/CAR-FoxP3, scFv/CAR-FoxP3, or CAR-FoxP3 alone. The cells were then sorted using anti-GD2 CAR staining and mCherry expression, and cultured for 8 weeks in IL-2-free RPMI complete media at 1 million /ml /well in 12-well plates. The viability of the cells was analyzed weekly by flow cytometry using DAPI staining. Also, 50% of the cultured media was replaced with new RPMI media weekly (scheme). The graph in the middle shows the percent viability of the three groups over eight weeks. The bottom is a representative overlaid histogram showing each group's DAPI-positive staining from week 1 to week 8. The two-way ANOVA multiple comparisons statistical analysis was performed using Prism 10. means ± SD; $n = 3$. (***$P < 0.001$).

 © The Author(s)
