# Supplementary material for: Targeted cellular micropharmacies deliver therapeutic agents to the brain
Source: EMBO Mol Med. 2026 Apr 14;18(6):2455–82. doi: 10.1038/s44321-026-00421-9 (PMC13270026; doi:10.1038/s44321-026-00421-9)
Supplement: Supplementary file 2 — Peer Review File [file 44321_2026_421_MOESM2_ESM.pdf]

EMBO Molecular Medicine

Targeted Cellular Micropharmacies Deliver Therapeutic Agents to the Brain

Manish Malviya, Subha Baniya, Eitan Wong, Tanya Jain, Branavan Manoranjan, Kristen Vogt, Zoe Kehs, Pedro Silberman, Tao Dao, Yueming Li, and David Scheinberg

Corresponding authors: David Scheinberg (scheinbd@mskcc.org) , Yueming Li (liy2@mskcc.org)

Review Timeline:

Submission Date:	19th Dec 25
Editorial Decision:	22nd Jan 26
Revision Received:	2nd Mar 26
Accepted:	17th Mar 26

Editor: Zeljko Durdevic

Transaction Report:

Please note that the manuscript was previously reviewed at another journal. As EMBO Press has a transfer agreement with that journal, revision was invited based on the reports from that previous external submission.

(Note: With the exception of the correction of typographical or spelling errors that could be a source of ambiguity, letters and reports are not edited. Depending on transfer agreements, referee reports obtained elsewhere may or may not be included in this compilation. Referee reports are anonymous unless the Referee chooses to sign their reports.)

Point by Point Response:

Reviewer #1 (Report for the authors (Required)):

The study by Manish Malviya and colleagues introduces a novel method to treat Alzheimer's disease (AD) and other neurodegenerative disorders. The developed approach enables CD4+ regulatory T cells (Treg) to effectively cross the blood-brain barrier, then deliver therapeutic cargos (such as antibodies) that promote improved clearance of amyloid beta (Aβ) or other disease-related proteins and pathways by enhancing microglial neurorestorative functions or phagocytosis. The approach outlined

in the study could be applied to a variety of other agents. Each or all could be delivered to a diseased brain using a replicative platform. The concept put forward by the authors and developed, in measure, within the current report centers on demonstrated abilities to engineer CD4+ Tregs to express chimeric antigen receptor (CAR) targeting GD2. This facilitated the entry of Treg cells into the brain. In the report, the cells were engineered to carry a CD33 antibody-blocking agent, which enhances microglial phagocytosis of Aβ. The developed idea is that a CAR that targets GD2 can direct CD4+ Tregs into the brain to seek neural target cells by using GD2 as a target surface marker. After the T-cells were engineered to express the GD2-specific CAR, they were administered to the animals. The GD2-CAR T-cells were shown to be capable of infiltrating the brain, recognizing and targeting the neuronal antigen GD2, and delivering their antibody "disease combating" cargo. The CD33 receptor-blocking effector was developed and tested. The blockade of this antigen enhanced microglial cells' ability to phagocytose Aβ plaques and, as a result, halt disease progression. Others acknowledge this process as a key feature of AD pathobiology. It is also well accepted that CD33 acts as an inhibitor of microglial phagocytosis, and its blockade reduces this inhibitory signal, thereby boosting the cell’s ability to clear Aβ. Antibodies targeting CD33 bind to the receptor, thereby enabling the best possible clearance of a key factor in the disease mechanism. This occurs as its binding has been shown to positively affect the internalization and degradation of CD33. The overarching idea is that blocking CD33 therapeutically restores the impaired microglial degradative capacity observed in AD pathobiology. The data presented in the current report, targeting CD33 expression, can not only reduce Aβ accumulation and inflammation through Treg cells but also halt disease progression. These findings indicate that therapeutic strategies targeting CD33 activity are a promising approach to enhance Aβ clearance. The investigators present a plethora of in vitro and ex vivo data showing that genetically engineered CAR Tregs targeting GD2 can direct the cells into the brain. Ectopic FoxP3 was selected by the research team to reduce inflammation, with secreted IL-2 promoting cell longevity. The idea is to use a form of CAR T-cell therapy previously developed to treat brain tumors to improve cell infiltration into the brain and enhance microglial phagocytic function.

Overall the proof of concept focus is to use ectopic FoxP3 to reduce inflammation, secreted IL-2 to promote cell longevity and secreted anti-CD33 antibody as a therapeutic agent.

The idea is an attractive one but suffers from its complexity, inherent toxicities, impractiable production scheme and translational utility. The missing significant link in the current report is to apply the idea to a AD mouse model and demonstrate that it is effective. The requested additional data is required to demonstrate that the system can be applied successfully in a relevant model of AD.

We agree that the cell construct we present is complex and currently possibly impractical for human application. However, this is an intractable disease with rare successes, and many new therapeutic ideas start out as difficult and unfeasible and as the field evolves other investigators improve and simplify the method. A good example is CAR T cells, invented in the late 1990s, but not made feasible for another decade. We provide the constructs as a promising proof of the concept, well elaborated by the reviewer, that this is an approach that should warrant continued study, not just in AD but in other CNS diseases, where penetration of the therapeutic agent is difficult. We will modify the discussion to address the comment.

The test in vivo is of course important. We spent nearly 2 years attempting to develop a usable model for AD in the mice in which to test this idea. We do not think this is possible and hence requesting this experiment before publication is not reasonable. The available mouse models cited or others that are proprietary do not solve the problem as the host mice need to be fully <u>immunocompromised</u> to allow engraftment of the engineered cells and retention of the humanized antibody to CD33, over long periods. Because the disease progresses over 4 to 8 months in the mice, we would have to maintain the cells in the mouse over this period or do repeated injections. This is not possible in immunocompetent mice where rejection of the cells occurs within days. Furthermore, to create a CD33+ x 5xAD+ x NSG+ model by crossing existing mice will require years; and to conduct the studies, if the mice ever become available, would require another 6-8 months <u>per experiment</u>. Therefore, even if feasible this would be several years of additional work. Unfortunately, these experiments are beyond the reach of our laboratories.

We have addressed these issues directly now in the discussion, to explain why the conduct of these in vivo studies, while important for the future, are not currently feasible.

Reviewer #2 (Report for the authors (Required)):

This manuscript describes a genetic tour-de-force in getting a cell into the brain to secrete an antibody-based therapeutic with potential for improved outcomes in neurodegeneration. While this approach is extremely proof-of-concept (likely much too complicates to make into a therapy), it provides the basis to explore further. The story is well written, experiments appear fairly rigorous, and there is a sound rational for the most part (see caveats on the target of choice and use of mouse models). Overall, this is a solid study but the authors should consider the following:

1) In the abstract it is written: “chimeric antigen receptor against GD2 to direct the cells

into the brain". This makes it sound like it helps get it across the blood brain barrier, which I believe is not what is trying to be evoked. Rather its retention in the CNS.

We have fixed this. The Introduction and Results also explicitly distinguish trafficking from retention.

2) A major flaw is the rational of targeting CD33. The authors themselves mention that CD33 in mouse is a bad model of CD33, and yet they reference papers that exactly mouse CD33 (Ref 19, 59). There are new papers that focus on human CD33 that are missed.

CD33 is targeted because it achieves the desired goal of increasing microglial uptake of AB plaque. The rationale is now refocused on human CD33 biology in microglia. Mouse CD33 citations are reframed to avoid implying mechanistic fidelity. New human-centric references are added, including Mol Neurodegener. 2024;19:42 (DOI: 10.1186/s13024-024-00734-8), the Biocytogen B-hCD33 resource (https://biocytogen.com/gene-humanized-models/b-hcd33-mice), and the AAIC conditional hCD33 KI abstract (DOI: 10.1002/alz.086747; Abstract ID 086747). We state species-specific differences and base mechanistic conclusions on human macrophage/microglia-derived data.

3) Similar to the point above, they conclude by stating that "suppression mechanism cannot be tested" in a mouse model, although a humanized CD33 mouse model is available and was not cited.

The test in vivo is of course important. The model is now cited and discussed. We acknowledge available humanized CD33 models (B-hCD33; conditional hCD33 KI). The available mouse models cited or others that are proprietary do not solve the problem as the host mice need to be fully immunocompromised to allow engraftment of the engineered cells and retention of the humanized antibody to CD33, over long periods. Because the disease progresses over 4 to 8 months in the mice, we would have to maintain the cells in the mouse over this period or do repeated injections. This is not possible in immunocompetent mice where rejection of the cells occurs within days. Furthermore, to create a CD33+ x 5xAD+ x NSG+ model by crossing existing mice will require years; and to conduct the studies, if the mice ever become available, would require another 6-8 months per experiment. Therefore, even if feasible this would be several years of additional work. Unfortunately, these experiments are beyond the reach of our laboratories.

We spent nearly 2 years attempting to develop a usable model for AD in the mice in which to test this idea. We do not think this is possible and hence requesting this experiment before publication is not reasonable.

We have addressed these issues directly now in the discussion, to explain why the conduct of these in vivo studies, while important for the future, are not currently feasible.

4) On Figure 2, the % phagocytic cells is mention in panel a on the graph but they then quantify based on MFI, which makes it appear that they are cherry-picking what to quantify. Would be better to be consider or show both.

This was re-analyzed and presented differently:

- *Percentage of pHrodo-positive cells (threshold defined using Latrunculin-A negative controls).*
- *MFI of pHrodo signal among positives.*
- *We now report % pHrodo+ (responder frequency), MFI within positives (per-cell uptake), and volume-normalized absolute pHrodo+ counts. This addresses the "cherry-picking" concern by showing both frequency and magnitude with uniform Latrunculin A–defined gating.*

Legends and Methods specify gating, thresholds, compensation, and statistical tests.

5) How were the total # of T cells in the brain calculated. Was a % collected and then extrapolated for the whole brain? Was this done in conjunction with counting beads? I could not find the experimental details.

We have added these details in a new section of the methods entitled: Absolute quantification of engineered T cells recovered from CNS compartments.

6) There should not a space between the numbers and units in the methods.

We have fixed this.

22nd Jan 2026

Dear Dr. Scheinberg,

Thank you for transferring your revised manuscript to EMBO Molecular Medicine and please accept my apologies for the delay in getting back to you due to the holiday season and large number of submissions we experienced recently. I am pleased to inform you that we will be able to accept your manuscript pending the following final amendments:

1) Authors: There is a discrepancy in how the name is displayed in our system (Malviya Manish) and in the manuscript (Manish Malviya), please correct.
2) Source data: Thank you for providing source data, however it appears that source data are missing for following figures:
- Fig1 Western blot, numerical data and flow cytometry FCS files.
- Fig2 numerical data and FCS files.
- Fig3 numerical data and FCS files. RNA seq raw data should be deposited in a public repository (see below).
- Fig4 numerical data, FCS files and microscopic images.
- Fig5 numerical data and FCS files.
- Fig6 numerical data and FCS files.
Please make sure to complete the source data checklist (attached to this email), provide all source data and upload them as one folder per figure with clearly labeled panels.
3) Figures:
- Please upload EV figures as individual high-resolution files. Also, although high resolution main figures appear pixelated, please correct. Please check "Author Guidelines" for more information:
https://www.embopress.org/page/journal/17574684/authorguide#figureformat
https://www.embopress.org/page/journal/17574684/authorguide#expandedview
- We note that some panels are reused between figures, e.g. Figure 4F panels are reused in EV Figure 8. Please cite in the respective figure legend every reused image/panel.
- In Figure 4B panels D7 CAR/Gluc and D21 CAR/FoxP3/Gluc are the same. Please explain and correct.
4) In the main manuscript file, please do the following:
- Please address all comments suggested by our data editors listed below:
o Figure legends:
1. Please note that the exact p values are not provided in the legends of figures 1D, 2A, B; 3D, 4E, 5D, 6E, F; EDF 1, EDF 2, EDF 4 C, EDF 5, EDF 7.
2. Please indicate the statistical test used for data analysis in the legends of figures 2A, 6E, F.
3. Please note that in figure 6B there is a mismatch between the annotated p values in the figure legend and the annotated p values in the figure file that should be corrected.
4. Please note that the box plots need to be defined in terms of minima, maxima, centre, bounds of box and whiskers, and percentile in the legends of figure 4E.
5. Please note that information related to n is missing in the legend of figure 6B.
6. Please note that the error bars are not defined in the legends of figures 5D, 6B, E, F.
7. Please note that the scale bar needs to be defined for figure 4F.
- The manuscript sections should be in the following order: Title page - Abstract & Keywords - Introduction - Results - Discussion - Methods - Data Availability - Acknowledgments - Disclosure Statement & Competing Interests - References - Figure Legends - (Main Tables with legends if applicable) - Expanded View Figure Legends.
- Figure callouts should be in sequential order. Currently Fig. 5 is called out before Fig. 4A, please correct. Also, please correct callouts of Suppl. fig.6 and Supplementary Figure 8 to Figure EV6 and EV8.
- In Methods, provide the antibody dilutions that were used for each antibody.
- Indicate in legends number and nature of replicates and exact p= values, not a range, along with the statistical test used. To keep the figures "clear" some authors found providing an Appendix table Sx with all exact p-values preferable. You are welcome to do this if you want to.
- Move data availability statement after Acknowledgments.
- Please rename "Competing Interests" to "Disclosure and competing interests statement". We updated our journal's competing interests policy in January 2022 and request authors to consider both actual and perceived competing interests. Please review the policy https://www.embopress.org/competing-interests and update your competing interests if necessary.
- In Disclosure and competing interests statement please state employment in a biotech company (affiliation #2).
- Author contributions: Please remove it from the manuscript and specify author contributions in our submission system. CRediT has replaced the traditional author contributions section because it offers a systematic machine-readable author contributions format that allows for more effective research assessment. You are encouraged to use the free text boxes beneath each contributing author's name to add specific details on the author's contribution. More information is available in our guide to authors:
https://www.embopress.org/page/journal/17574684/authorguide#authorshipguidelines
- In data availability statement remove the current sentence and provide information about raw data from large-scale datasets like RNA-seq that should be deposited in one of the relevant databases and made freely available prior the publication of the

manuscript. Use the following format to report the accession number of your data:

The datasets produced in this study are available in the following databases:
[data type]: [full name of the resource] [accession number/identifier] ([doi or URL or identifiers.org/DATABASE:ACCESSION])
- Remove "Graphical presentation" from the figure legends. This should be incorporated in the synopsis text (see below).
- Correct the reference citation in the text and reference list. In the text, a reference should be cited by author and year of publication. Include a space between a word and the opening parenthesis of the reference that follows. In the reference list, citations should be listed in alphabetical order. Where there are more than 10 authors on a paper, 10 will be listed, followed by "et al.". Please check "Author Guidelines" for more information.
https://www.embopress.org/page/journal/17574684/authorguide#referencesformat
5) Funding: Please rename it to Acknowledgements. Make sure that information about all sources of funding are complete in both our submission system and in the manuscript. Currently the following funding sources are missing in our submission system: RO1 CA55349, PO1 CA23766, the Sunshine Fund, R01AG080684, the JPB Foundation, Cure Alzheimer's Fund, BrightFocus Foundation, MSK Cancer Center Support Grant/Core Grant (Grant P30 CA008748), The Cycle for Survival, Mr. William H. Goodwin and Mrs. Alice Goodwin and the Commonwealth Foundation for Cancer Research, the Experimental Therapeutics Center of MSKCC, and the William Randolph Hearst Fund in Experimental Therapeutics, National Science Foundation Graduate Research Fellowship 1746886 and National Institutes of Health T32 grants GM115327 & GM136640. The missing funders should be provided as separate entries via More Funders option (Comments box should not be used).
6) The Paper Explained: Please provide "The Paper Explained" and add it to the main manuscript text. Please check "Author Guidelines" for more information. https://www.embopress.org/page/journal/17574684/authorguide#researcharticleguide
7) Synopsis:
- Synopsis image: Please upload the image as a high-resolution .jpeg/.png file 550 px-wide x 300-600 pixels high.
- Synopsis text: Please provide a short standfirst (maximum of 300 characters, including space) as well as 2-5 one sentence bullet points that summarise the paper as a .doc file. Please write the bullet points to summarise the key NEW findings. They should be designed to be complementary to the abstract - i.e. not repeat the same text. We encourage inclusion of key acronyms and quantitative information (maximum of 30 words / bullet point). Please use the passive voice.
- Please check your synopsis text and image before submission with your revised manuscript. Please be aware that in the proof stage minor corrections only are allowed (e.g., typos).
8) As part of the EMBO Publications transparent editorial process (see our Editorial at http://embomolmed.embopress.org/content/2/9/329), EMBO Molecular Medicine will publish online a Review Process File (RPF) to accompany accepted manuscripts. This file will be published in conjunction with your paper and will include the anonymous referee reports, your point-by-point response and all pertinent correspondence relating to the manuscript. Let us know if you want to remove or not any figures from it prior to publication. Please note that the Authors checklist will be published at the end of the RPF.
9) Please provide a point-by-point letter to my comments (as Word file).

I look forward to reading a new revised version of your manuscript as soon as possible.

Yours sincerely,

Zeljko Durdevic

Zeljko Durdevic
Senior Editor
EMBO Molecular Medicine

*** Instructions to submit your revised manuscript ***

*** PLEASE NOTE *** As part of the EMBO Publications transparent editorial process initiative (see our Editorial at https://www.embopress.org/doi/pdf/10.1002/emmm.201000094), EMBO Molecular Medicine will publish online a Review Process File to accompany accepted manuscripts.

In the event of acceptance, this file will be published in conjunction with your paper and will include the anonymous referee reports, your point-by-point response and all pertinent correspondence relating to the manuscript. If you do NOT want this file to be published, please inform the editorial office at contact@embomolmed.org.

*****

When preparing your revised manuscript, please refer to our guidelines: https://link.springer.com/journal/44321/submission-guidelines#cms-Revised-submissions. We perform an initial quality control of all revised manuscripts before re-review; failure to include requested items will delay the evaluation of your revision.

We require:

1) A .docx formatted version of the manuscript text (including legends for main figures, EV figures and tables). Please make sure that the changes are highlighted to be clearly visible.

2) Individual production quality figure files as .eps, .tif, .jpg (one file per figure). For guidance, download the 'Figure Guide PDF': https://media.springernature.com/original/springer-cms/rest/v1/content/27825798/data/v1.

3) A .docx formatted letter INCLUDING the reviewers' reports and your detailed point-by-point responses to their comments. As part of the EMBO Press transparent editorial process, the point-by-point response is part of the Review Process File (RPF), which will be published alongside your paper.

4) A complete author checklist, which you can download from our author guidelines. Please insert information in the checklist that is also reflected in the manuscript. The completed author checklist will also be part of the RPF.

5) Please note that all corresponding authors are required to supply an ORCID ID for their name upon submission of a revised manuscript.

6) It is mandatory to include a 'Data Availability' section after the Materials and Methods. Before submitting your revision, primary datasets produced in this study need to be deposited in an appropriate public database, and the accession numbers and database listed under 'Data Availability'. Please remember to provide a reviewer password if the datasets are not yet public.

In case you have no data that requires deposition in a public database, please state so in this section. Note that the Data Availability Section is restricted to new primary data that are part of this study.

7) For data quantification: please specify the name of the statistical test used to generate error bars and P values, the number (n) of independent experiments (specify technical or biological replicates) underlying each data point and the test used to calculate p-values in each figure legend. The figure legends should contain a basic description of n, P and the test applied. Graphs must include a description of the bars and the error bars (s.d., s.e.m.).

8) At EMBO Press we ask authors to provide source data for the main manuscript figures. You will receive a separate email with instructions for providing source data with your revised manuscript, including how to upload and organize the files.

9) Our journal encourages inclusion of *data citations in the reference list* to directly cite datasets that were re-used and obtained from public databases. Data citations in the article text are distinct from normal bibliographical citations and should directly link to the database records from which the data can be accessed. In the main text, data citations are formatted as follows: "Data ref: Smith et al, 2001" or "Data ref: NCBI Sequence Read Archive PRJNA342805, 2017". In the Reference list, data citations must be labeled with "[DATASET]". A data reference must provide the database name, accession number/identifiers and a resolvable link to the landing page from which the data can be accessed at the end of the reference.

10) We replaced Supplementary Information with Expanded View (EV) Figures and Tables that are collapsible/expandable online. A maximum of 5 EV Figures can be typeset. EV Figures should be cited as 'Figure EV1, Figure EV2" etc... in the text and their respective legends should be included in the main text after the legends of regular figures.

- For the figures that you do NOT wish to display as Expanded View figures, they should be bundled together with their legends in a single PDF file called *Appendix*, which should start with a short Table of Content. Appendix figures should be referred to in the main text as: "Appendix Figure S1, Appendix Figure S2" etc.

- Additional Tables/Datasets should be labeled and referred to as Table EV1, Dataset EV1, etc. Legends have to be provided in a separate tab in case of .xls files. Alternatively, the legend can be supplied as a separate text file (README) and zipped together with the Table/Dataset file.

11) The paper explained: EMBO Molecular Medicine articles are accompanied by a summary of the articles to emphasize the major findings in the paper and their medical implications for the non-specialist reader. Please provide a draft summary of your article highlighting

- the medical issue you are addressing,

- the results obtained and

- their clinical impact.

This may be edited to ensure that readers understand the significance and context of the research. Please refer to any of our published articles for an example.

12) Author contributions: You will be asked to provide CRediT (Contributor Role Taxonomy) terms in the submission system. These replace a narrative author contribution section in the manuscript.

13) A Conflict of Interest statement should be provided in the main text.

14) Every published paper includes a 'Synopsis' to further enhance discoverability. Synopses are displayed on the journal webpage and are freely accessible to all readers. They include a short stand first (maximum of 300 characters, including space) as well as 2-5 one-sentences bullet points that summarizes the paper. Please write the bullet points to summarize the key NEW findings. They should be designed to be complementary to the abstract - i.e. not repeat the same text. We encourage inclusion of key acronyms and quantitative information (maximum of 30 words / bullet point). Please use the passive voice. Please attach these in a separate file or send them by email, we will incorporate them accordingly.

Please also suggest a striking image or visual abstract to illustrate your article as a PNG file 550 px wide x 300-600 px high.

15) Include a Reagents and Tools Table as part of the Methods section, which can be downloaded from our author guidelines.

EMBO Molecular Medicine has a "scooping protection" policy, whereby similar findings that are published by others during review or revision are not a criterion for rejection. Should you decide to submit a revised version, I do ask that you get in touch after three months if you have not completed it, to update us on the status.

*Additional important information regarding Figures

Each figure should be given in a separate file and should have the following resolution:
Graphs 800-1,200 DPI
Photos 400-800 DPI
Colour (only CMYK) 300-400 DPI"

Figures are not edited by the production team. All lettering should be the same size and style; figure panels should be indicated by capital letters (A, B, C etc). Gridlines are not allowed except for log plots. Figures should be numbered in the order of their appearance in the text with Arabic numerals. Each Figure must have a separate legend and a caption is needed for each panel.

*Additional important information regarding figures and illustrations can be found at
https://media.springernature.com/original/springer-cms/rest/v1/content/27825798/data/v1

The authors addressed the remaining editorial issues.

17th Mar 2026

Dear Dr. Scheinberg,

We are pleased to inform you that your manuscript is accepted for publication and is now being sent to our publisher to be included in the next available issue of EMBO Molecular Medicine.

Your manuscript will be processed for publication by EMBO Press. It will be copy edited and you will receive page proofs prior to publication. Please note that you will be contacted by Springer Nature Author Services to complete licensing and payment information.

You may qualify for financial assistance for your publication charges - either via a Springer Nature fully open access agreement or an EMBO initiative. Check your eligibility: https://link.springer.com/journal/44321/how-to-publish-with-us

Should you be planning a Press Release on your article, please get in contact with embo_production@springernature.com as early as possible in order to coordinate publication and release dates.

If you have any questions, please do not hesitate to contact the Editorial Office. Thank you for your contribution to EMBO Molecular Medicine.

Yours sincerely,
Zeljko Durdevic

Zeljko Durdevic
Senior Editor
EMBO Molecular Medicine

>>> Please note that it is EMBO Molecular Medicine policy for the transcript of the editorial process (containing referee reports and your response letter) to be published as an online supplement to each paper. If you do NOT want this, you will need to inform the Editorial Office via email immediately. More information is available here: https://link.springer.com/partners/embo-press/editorial-policies#Peer%20review

EMBO Press Author Checklist

Corresponding Author Name: David A. Scheinberg	
Journal Submitted to: The EMBO Journal	
Manuscript Number: EMM-2025-23148	

USEFUL LINKS FOR COMPLETING THIS FORM
The EMBO Journal - Author Guidelines
EMBO Reports - Author Guidelines
Molecular Systems Biology - Author Guidelines
EMBO Molecular Medicine - Author Guidelines

Reporting Checklist for Life Science Articles (updated January
This checklist is adapted from Materials Design Analysis Reporting (MDAR) Checklist for Authors. MDAR establishes a minimum set of requirements in transparent reporting in the life sciences (see Statement of Task: 10.31222/osf.io/9sm4x). Please follow the journal's guidelines in preparing your
Please note that a copy of this checklist will be published alongside your article.

Abridged guidelines for figures
1. Data
The data shown in figures should satisfy the following conditions:
- the data were obtained and processed according to the field's best practice and are presented to reflect the results of the experiments in an accurate and unbiased manner.
- ideally, figure panels should include only measurements that are directly comparable to each other and obtained with the same assay.
- plots include clearly labeled error bars for independent experiments and sample sizes. Unless justified, error bars should not be shown for technical
- if n<5, the individual data points from each experiment should be plotted. Any statistical test employed should be justified.
- Source Data should be included to report the data underlying figures according to the guidelines set out in the authorship guidelines on Data

2. Captions
Each figure caption should contain the following information, for each panel where they are relevant:
- a specification of the experimental system investigated (eg cell line, species name).
- the assay(s) and method(s) used to carry out the reported observations and measurements.
- an explicit mention of the biological and chemical entity(ies) that are being measured.
- an explicit mention of the biological and chemical entity(ies) that are altered/varied/perturbed in a controlled manner.
- the exact sample size (n) for each experimental group/condition, given as a number, not a range;
- a description of the sample collection allowing the reader to understand whether the samples represent technical or biological replicates (including how many animals, litters, cultures, etc.).
- a statement of how many times the experiment shown was independently replicated in the laboratory.
- definitions of statistical methods and measures:
  - common tests, such as t-test (please specify whether paired vs. unpaired), simple $\chi 2$ tests, Wilcoxon and Mann-Whitney tests, can be unambiguously identified by name only, but more complex techniques should be described in the methods section;
  - are tests one-sided or two-sided?
  - are there adjustments for multiple comparisons?
  - exact statistical test results, e.g., P values = x but not P values < x;
  - definition of 'center values' as median or average;
  - definition of error bars as s.d. or s.e.m.

Please complete ALL of the questions below.
Select "Not Applicable" only when the requested information is not relevant for your study.

Materials

Newly Created Materials	Information included in the manuscript?	In which section is the information available? (Reagents and Tools Table, Materials and Methods, Figures, Data Availability Section)
New materials and reagents need to be available; do any restrictions apply?	Not Applicable	Unpublished sequences pending IP review will become available upon protection.

Antibodies	Information included in the manuscript?	In which section is the information available? (Reagents and Tools Table, Materials and Methods, Figures, Data Availability Section)
For **antibodies** provide the following information:<br>- Commercial antibodies: RRID (if possible) or supplier name, catalogue number and or/clone number<br>- Non-commercial: RRID or citation	Yes	Materials and Methods

DNA and RNA sequences	Information included in the manuscript?	In which section is the information available? (Reagents and Tools Table, Materials and Methods, Figures, Data Availability Section)
**Short novel DNA or RNA including primers, probes**: provide the sequences.	Not Applicable	Will be posted in online data sharing site or available from the authors after publication.

Cell materials	Information included in the manuscript?	In which section is the information available? (Reagents and Tools Table, Materials and Methods, Figures, Data Availability Section)
**Cell lines:** Provide species information, strain. Provide accession number in repository **OR** supplier name, catalog number, clone number, and/**OR** RRID.	Yes	Materials and Methods
**Primary cultures:** Provide species, strain, sex of origin, genetic modification status.	Yes	Materials and Methods
Report if the cell lines were recently **authenticated** (e.g., by STR profiling) and tested for mycoplasma contamination.	Yes	Materials and Methods

Experimental animals	Information included in the manuscript?	In which section is the information available? (Reagents and Tools Table, Materials and Methods, Figures, Data Availability Section)
**Laboratory animals or Model organisms:** Provide species, strain, sex, age, genetic modification status. Provide accession number in repository **OR** supplier name, catalog number, clone number, **OR** RRID.	Yes	Materials and Methods
**Animal observed in or captured from the field:** Provide species, sex, and age where possible.	Not Applicable	None
Please detail **housing and husbandry conditions**.	Yes	Materials and Methods

Plants and microbes	Information included in the manuscript?	In which section is the information available? (Reagents and Tools Table, Materials and Methods, Figures, Data Availability Section)
**Plants:** provide species and strain, ecotype and cultivar where relevant, unique accession number if available, and source (including location for collected wild specimens).	Not Applicable	N/A
**Microbes:** provide species and strain, unique accession number if available, and source.	Not Applicable	N/A

Human research participants	Information included in the manuscript?	In which section is the information available? (Reagents and Tools Table, Materials and Methods, Figures, Data Availability Section)
If collected and within the bounds of privacy constraints report on age, sex and gender or ethnicity for all study participants.	Not Applicable	Materials and Methods

Core facilities	Information included in the manuscript?	In which section is the information available? (Reagents and Tools Table, Materials and Methods, Figures, Data Availability Section)
If your work benefited from core facilities, was their service mentioned in the acknowledgments section?	Yes	Materials and Methods

Design

Study protocol	Information included in the manuscript?	In which section is the information available? (Reagents and Tools Table, Materials and Methods, Figures, Data Availability Section)
If study protocol has been **pre-registered, provide DOI in the manuscript**. For clinical trials, provide the trial registration number **OR** cite DOI.	Not Applicable	N/A
Report the **clinical trial registration number** (at ClinicalTrials.gov or equivalent), where applicable.	Not Applicable	N/A

Laboratory protocol	Information included in the manuscript?	In which section is the information available? (Reagents and Tools Table, Materials and Methods, Figures, Data Availability Section)
Provide DOI OR other citation details if **external detailed step-by-step protocols** are available.	Yes	Materials and Methods

Experimental study design and statistics	Information included in the manuscript?	In which section is the information available? (Reagents and Tools Table, Materials and Methods, Figures, Data Availability Section)
Include a statement about **sample size** estimate even if no statistical methods were used.	Not Applicable	N/A
Were any steps taken to minimize the effects of subjective bias when allocating animals/samples to treatment (e.g. **randomization procedure**)? If yes, have they been described?	Yes	Materials and Methods
Include a statement about **blinding** even if no blinding was done.	Not Applicable	N/A
Describe **inclusion/exclusion criteria** if samples or animals were excluded from the analysis. Were the criteria pre-established?<br><br>If sample or data points were omitted from analysis, report if this was due to attrition or intentional exclusion and provide justification.	Not Applicable	N/A
For every figure, are **statistical tests** justified as appropriate? Do the data meet the assumptions of the tests (e.g., normal distribution)? Describe any methods used to assess it. Is there an estimate of variation within each group of data? Is the variance similar between the groups that are being statistically compared?	Yes	Materials and Methods, Figure legends

Sample definition and in-laboratory replication	Information included in the manuscript?	In which section is the information available? (Reagents and Tools Table, Materials and Methods, Figures, Data Availability Section)
In the figure legends: state number of times the experiment was **replicated** in laboratory.	Yes	Figure legends
In the figure legends: define whether data describe **technical or biological replicates**.	Yes	Figure legends

Ethics

Ethics	Information included in the manuscript?	In which section is the information available? (Reagents and Tools Table, Materials and Methods, Figures, Data Availability Section)
Studies involving **human participants**: State details of **authority granting ethics approval** (IRB or equivalent committee(s), provide reference number for approval.	Not Applicable	N/A
Studies involving **human participants**: Include a statement confirming that **informed consent** was obtained from all subjects and that the experiments conformed to the principles set out in the WMA Declaration of Helsinki and the Department of Health and Human Services Belmont Report.	Not Applicable	N/A
Studies involving **human participants: ** For publication of **patient photos**, include a statement confirming that consent to publish was obtained.	Not Applicable	N/A
Studies involving experimental **animals**: State details of **authority granting ethics approval** (IRB or equivalent committee(s), provide reference number for approval. Include a statement of compliance with ethical regulations.	Yes	Materials and methods
Studies involving **specimen and field samples:** State if relevant **permits** obtained, provide details of authority approving study; if none were required, explain why.	Not Applicable	N/A

Dual Use Research of Concern (DURC)	Information included in the manuscript?	In which section is the information available? (Reagents and Tools Table, Materials and Methods, Figures, Data Availability Section)
Could your study fall under dual use research restrictions? Please check biosecurity documents and list of **select agents and toxins** (CDC): https://www.selectagents.gov/sat/list.htm	Not Applicable	N/A
If you used a select agent, is the security level of the lab appropriate and reported in the manuscript?	Not Applicable	N/A
If a study is subject to dual use research of concern regulations, is the name of the **authority granting approval and reference number** for the regulatory approval provided in the manuscript?	Not Applicable	N/A

Reporting

The MDAR framework recommends adoption of discipline-specific guidelines, established and endorsed through community initiatives. Journals have their own policy about requiring specific guidelines and recommendations to complement MDAR.

Adherence to community standards	Information included in the manuscript?	In which section is the information available? (Reagents and Tools Table, Materials and Methods, Figures, Data Availability Section)
State if relevant guidelines or checklists (e.g., **ICMJE, MIBBI, ARRIVE, PRISMA**) have been followed or provided.	Not Applicable	none
For **tumor marker prognostic studies,** we recommend that you follow the **REMARK** reporting guidelines (see link list at top right). See author guidelines, under 'Reporting Guidelines'. Please confirm you have followed these guidelines.	Not Applicable	N/A
For **phase II and III randomized controlled trials**, please refer to the **CONSORT** flow diagram (see link list at top right) and submit the CONSORT checklist (see link list at top right) with your submission. See author guidelines, under 'Reporting Guidelines'. Please confirm you have submitted this list.	Not Applicable	N/A

Data Availability

Data availability	Information included in the manuscript?	In which section is the information available? (Reagents and Tools Table, Materials and Methods, Figures, Data Availability Section)
Have **primary datasets** been deposited according to the journal's guidelines (see 'Data Deposition' section) and the respective accession numbers provided in the Data Availability Section?	Yes	Will be posted in online data sharing site or available from the authors after publication.
Were **human clinical and genomic datasets** deposited in a public access-controlled repository in accordance to ethical obligations to the patients and to the applicable consent agreement?	Not Applicable	N/A
Are **computational models** that are central and integral to a study available without restrictions in a machine-readable form? Were the relevant accession numbers or links provided?	Not Applicable	N/A
If publicly available data were reused, provide the respective **data citations in the reference list**.	Not Applicable	NH/A
